# Supplementary material for: Ultra-fast switching memristors based on two-dimensional materials
Source: Nat Commun. 2024 Mar 14;15:2334. doi: 10.1038/s41467-024-46372-y (PMC10940724; doi:10.1038/s41467-024-46372-y)
Supplement: Supplementary file 1 — Supplementary Information [file 41467_2024_46372_MOESM1_ESM.pdf]

Ultra-fast switching memristors based on two-dimensional materials

SS Teja Nibhanupudi[1], Anupam Roy[1,2], Dmitry Veksler[3], Matthew Coupin[4], Kevin C. Matthews[4], Matthew Disiena[1], Ansh[1], Jatin V Singh[1], Ioana R Gearba-Dolocan[4], Jamie Warner[4], Jaydeep P. Kulkarni[1], Gennadi Bersuker[5], Sanjay K. Banerjee[1]

[1]Microelectronics Research Center, The University of Texas at Austin, Austin, Texas, 78758, USA

[2]Birla Institute of Technology, Mesra, Ranchi, 835215, India

[3]HRL Laboratories, Malibu, California, 90265, USA

[4]Texas Materials Institute, The University of Texas at Austin, 78712, USA

[5]M2D solutions, Austin, Texas, 78758, USA

Supplementary information

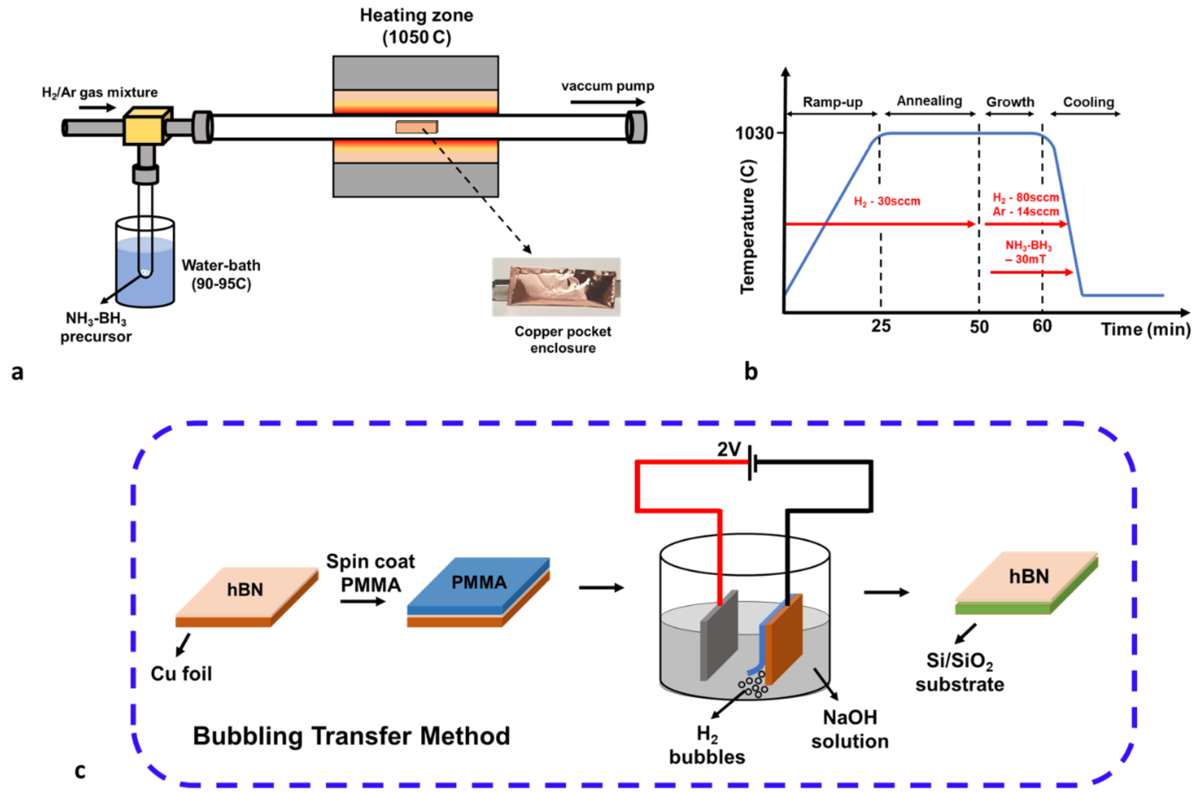

Supplementary Figure1: (a) Schematic of low pressure CVD growth chamber used for hBN synthesis on copper foils **(b)** Temperature profile of the furnace during the growth process **(c)** Schematic of the bubbling transfer method.

Few layer hBN stacks are grown by Chemical Vapor deposition (CVD) on copper foils [S1-S3] that are electropolished [S4-S5] to reduce surface roughness. The growth is conducted at 1030C using ammonia borane precursor [S1-S4]. The copper foil is folded into a pocket that creates a copper-saturated environment on the inside which limits the sublimation of copper, resulting in a smoother surface [S6-S7]. The smooth surface reduces the nucleation density promoting large-area growth with reduced grain boundaries. Grain boundaries can be detrimental to memristor performance since they accelerate ion migration and reduce the endurance of the device. The base pressure of the CVD system is 6mT before the growth. During annealing, the pressure reaches around 230mT with 30sccm H_2 flowrate. The pressure is increased to 650mT at the beginning of the growth with 80sccm H_2 and 14sccm Ar. A valve separates the main chamber from the precursor ampoule, as shown in Supplementary Fig. 1a. The ammonia borane decomposes when heated, and the pressure builds up within the ampoule. To ensure uniform growth pressure, the valve to the main chamber is opened only after the pressure within the ampoule reaches 50mT. This pressure is maintained throughout the growth duration. The grown hBN films were transferred from copper foil onto Si/SiO_2 (285nm) substrate through the bubbling transfer method [S8-S9].

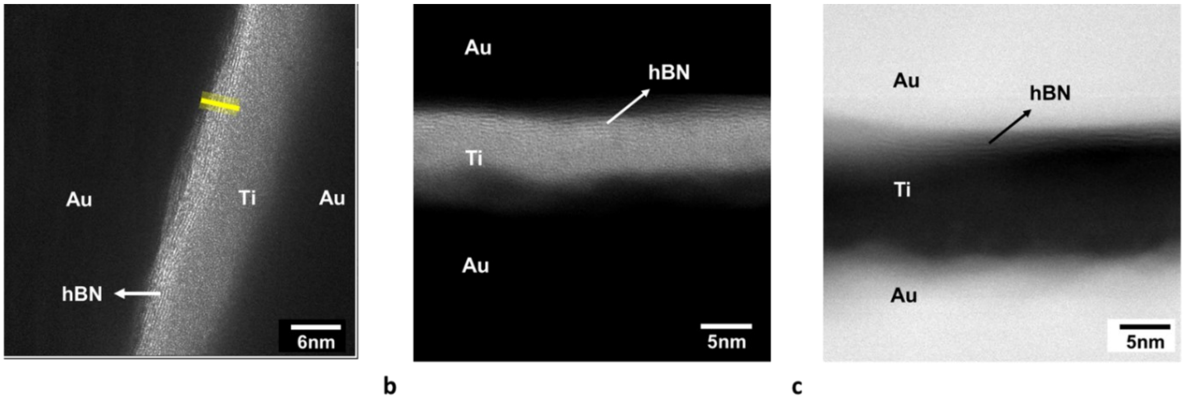

a b c

Supplementary figure 2: (a) Phase contrast TEM image of the memristor device cross-section showing the layered structure of hBN films. The titanium layer thickness matches with the crystal thickness monitor reading during e-beam evaporation (b) Annular Bright field scanning TEM image of the same FIB cross-section shows the layered structure, thereby confirming that the layers observed in phase contrast TEM image are not Fresnel fringes but originate from the hBN film (c) Annular Dark field scanning TEM (ADF-STEM) image of the same cross-section.

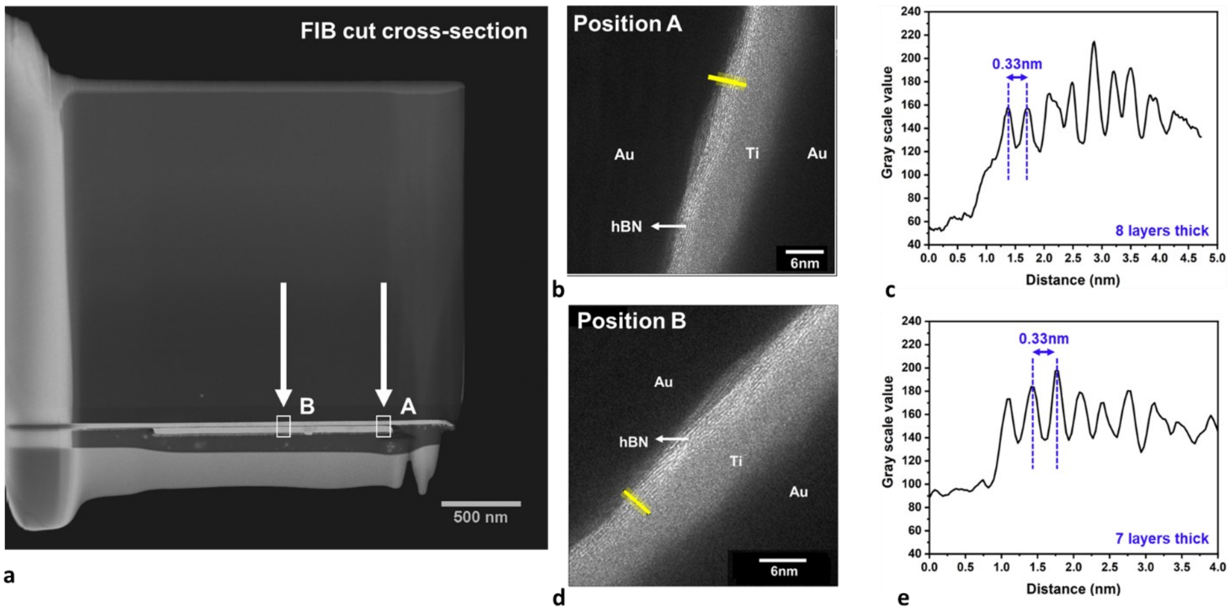

Supplementary figure 3: (a) Focused ion beam (FIB) cut cross-section of the sample highlighting two different locations A, B where the TEM images have been collected (b) Phase contrast TEM image from location A showing the layered structure (c) Grayscale pixel profile of the image across the marked yellow line shown in panel b. The grayscale profile shows 8 peaks corresponding to 8 hBN layers. The interlayer separation is around 0.33nm which closely matches reported values [S10-S12]. (d) Phase contrast TEM image from location B showing (e) Grayscale profile across the marked yellow line in panel d. The hBN thickness is around 7 layers.

The layered structure observed at different locations shows excellent uniformity of the synthesized hBN films.

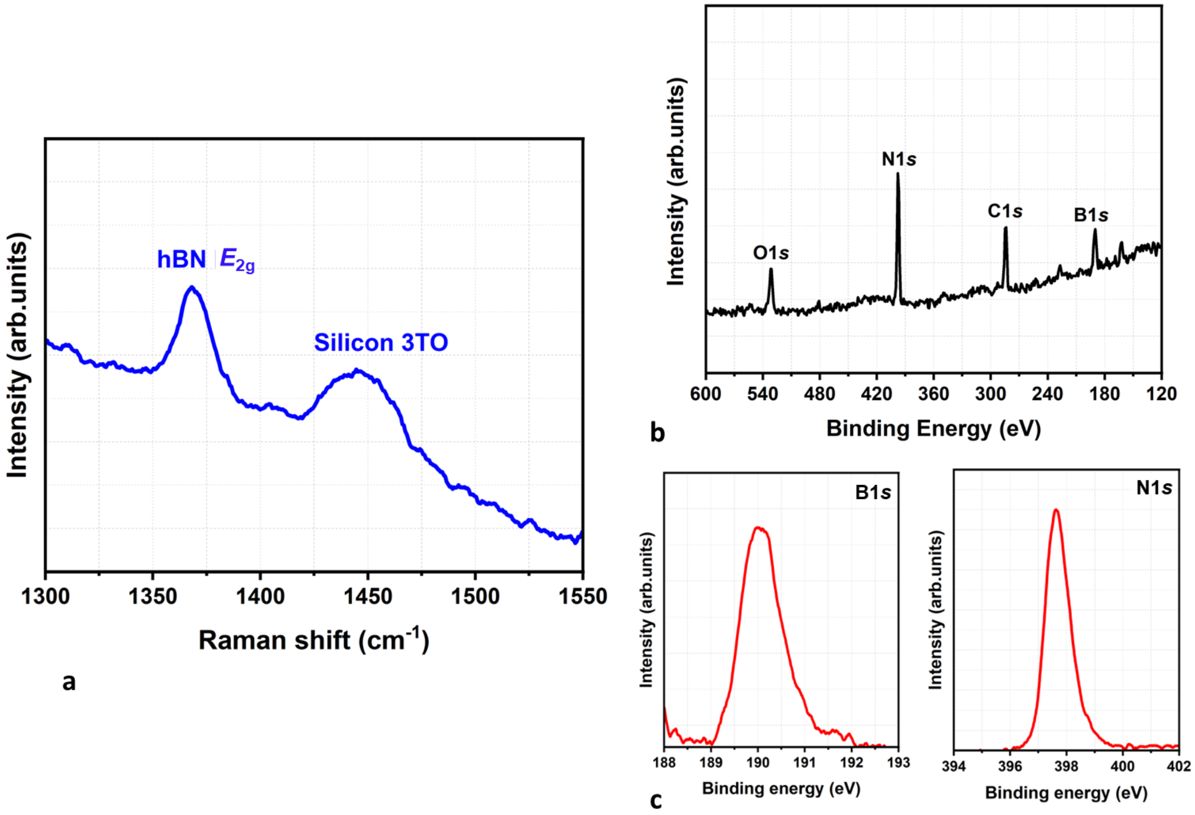

Supplementary figure 4: (a) Raman spectrum of the hBN film transferred onto Si/SiO_2 substrate. The E_{2g} peak at 1368cm^{-1} [S13-S14] confirms the good quality of the synthesized hBN films. (b) X-ray Photoelectron spectra of the transferred hBN film. The survey plot shows the elemental 1*s* core level peaks of B, C, N and O. (c) High-resolution XPS spectra collected around B 1*s* (190.2eV) and N 1s (397.7eV) [S15].

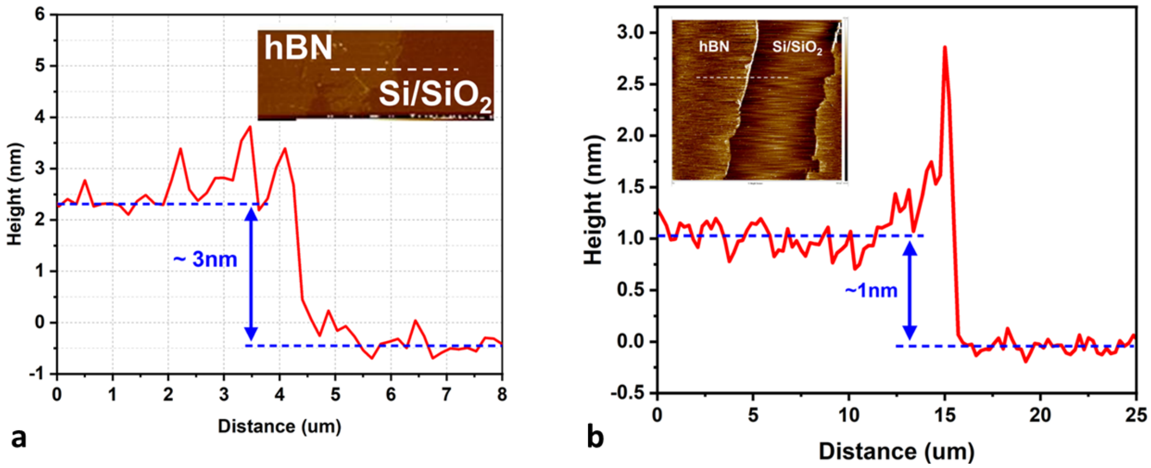

Supplementary figure 5: (a) Atomic force micrography (AFM) height profile of thick hBN (7-8 layers) across the line marked in the inset. The measured thickness of 3nm closely matches expected thickness of 8 layers hBN film (b) AFM height profile of the thin hBN (3-4 layers) across the line marked in the inset. The thin hBN films are synthesized under lower precursor vapor pressure conditions (P ~ 5mT) achieved by reducing the quantity of precursor added to the ampoule.

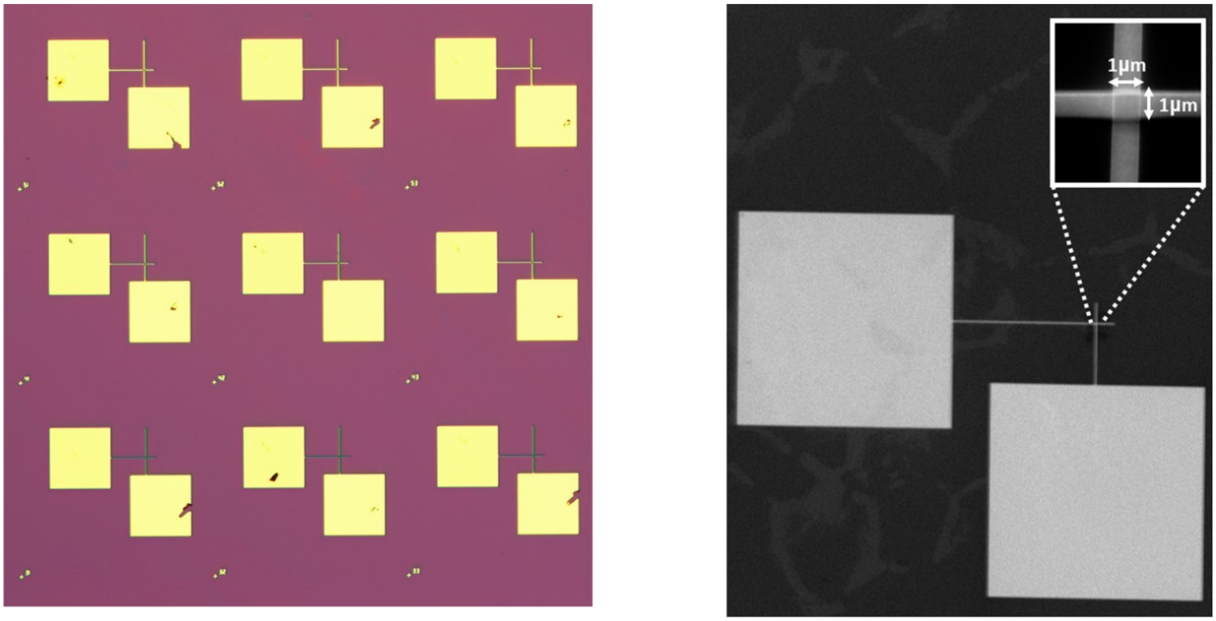

a b

Supplementary figure 6: (a) Optical Microscope image of the devices with varying cross-sectional dimensions (500x500nm^2 to 2x2um^2) (b) Scanning Electron Microscope image of the memristor device cross over point (inset) where top and bottom electrodes overlap.

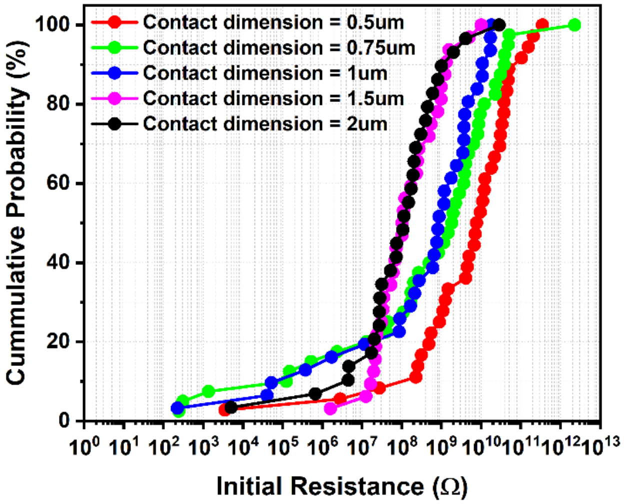

Supplementary figure 7.1: Cumulative distribution of Initial Resistance for devices with varying contact dimensions (0.5um shown in red, 0.75um shown in green, 1um shown in blue, 1.5um shown in pink, 2um shown in black). The initial resistance distribution shift to lower values as the device dimension increases.

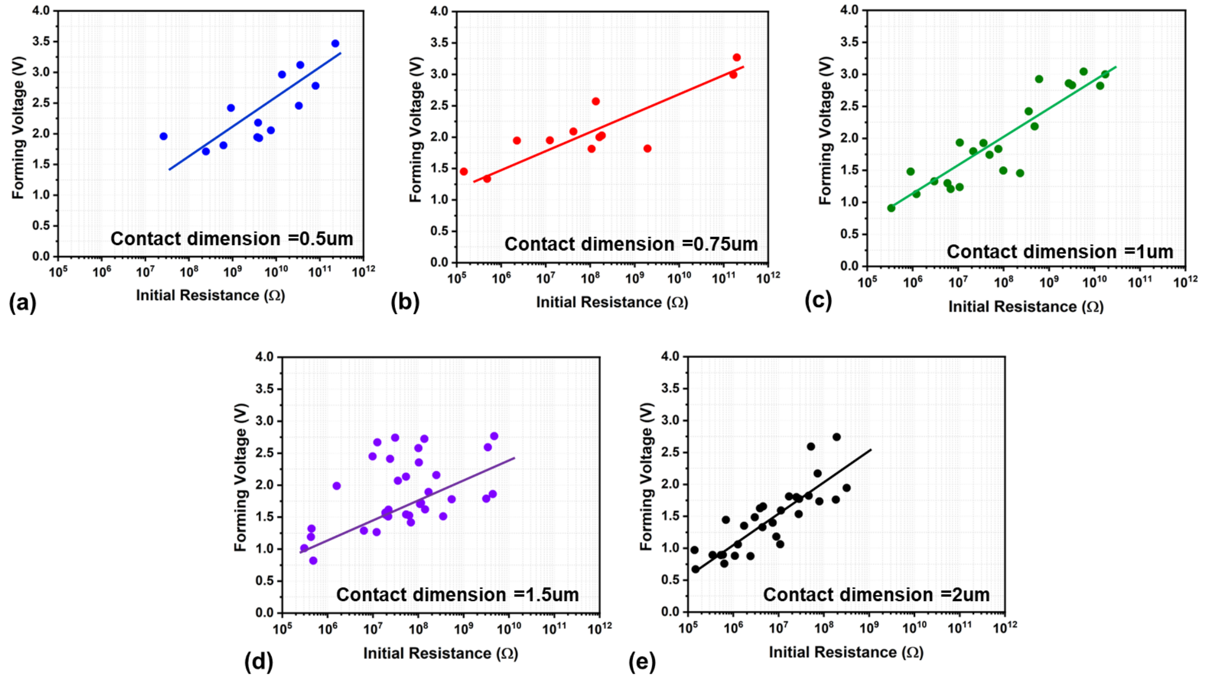

Supplementary figure 7.2: Forming voltage vs Initial Resistance for devices with contact dimensions (a) 0.5um (b) 0.75um (c) 1um (d) 1.5um (e) 2um. Evidently, the forming voltage increases with initial resistance for all device dimensions. Therefore, a generic relationship can be established between initial resistance and forming voltage for the 2D hBN based memristor devices.

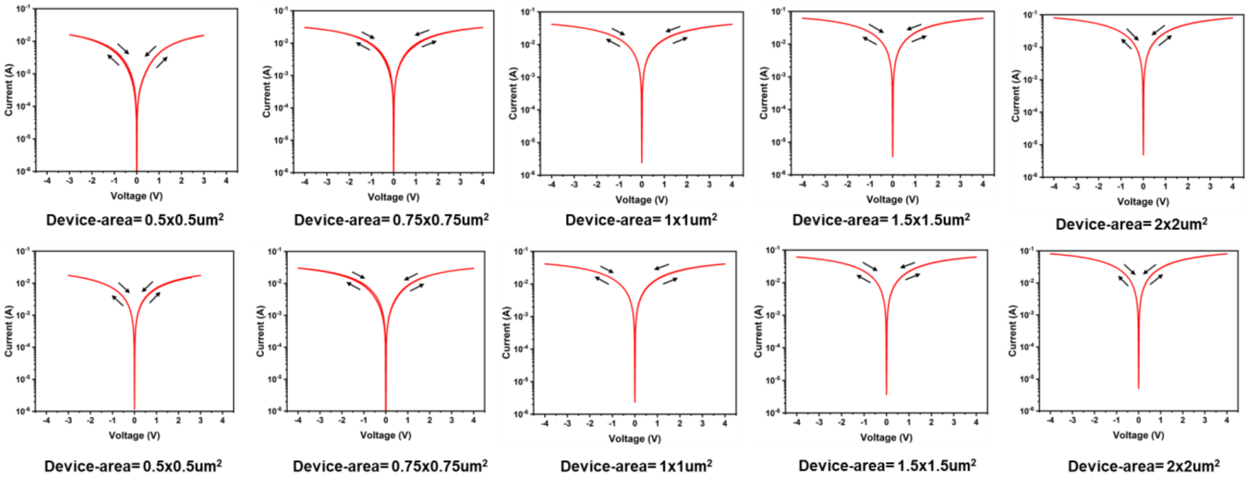

Supplementary figure 8: DC IV characteristics of Ti/Au devices (no hBN layer) with varying dimensions. All the devices exhibit ohmic conduction with no conceivable memory window. Moreover, the resistance of these devices is very low (50Ω -180Ω) compared to the devices with hBN (92% of devices with resistance > 1MΩ). Therefore, we can ascertain that the resistive switching observed in this study can be solely attributed to the hBN layer.

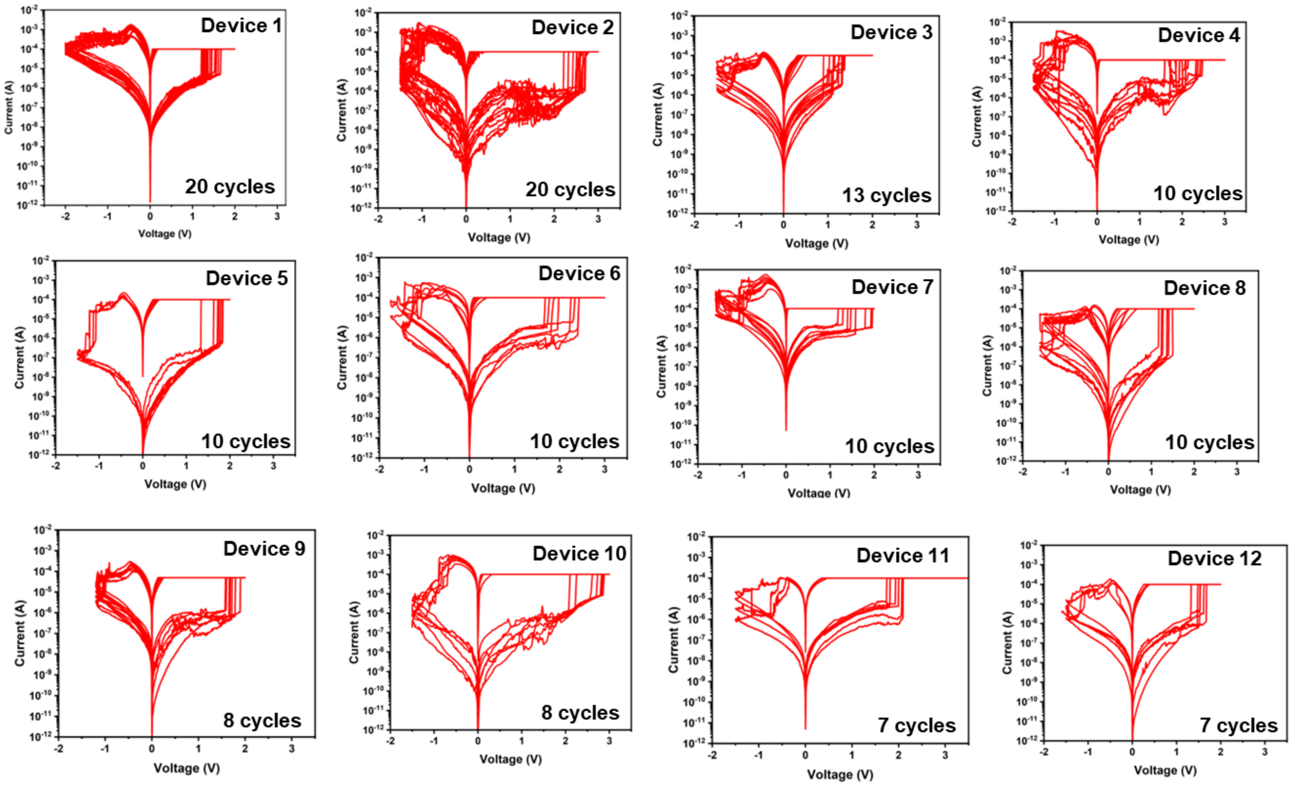

Supplementary figure 9: DC IV characteristics collected from 12 different devices with a current compliance limit of 100uA.

Variation in the device characteristics

The voltage distribution presented in Fig.2c of the main text shows a larger variation for SET (1V to 3V) than RESET. To understand the source of this variation, we segregated the data to cycle-to-cycle and device-to-device components. From Supplementary figure.9, qualitatively, we observe that the cycle-to-cycle variation is lower than the device-to-device variation. To quantify this observation, we chose four devices and plotted their IV characteristics in Supplementary figure.10. Here, although the mean SET voltage varies considerably (between 1.2V and 2.54V), the distributions are relatively tightly bound around the mean value with a worst-case standard deviation of 0.25V. Whereas the standard deviation of the overall distribution (Fig.2c) is about 0.5V. This indicates that the device-to-device variation is the dominant cause of variation for these memristors.

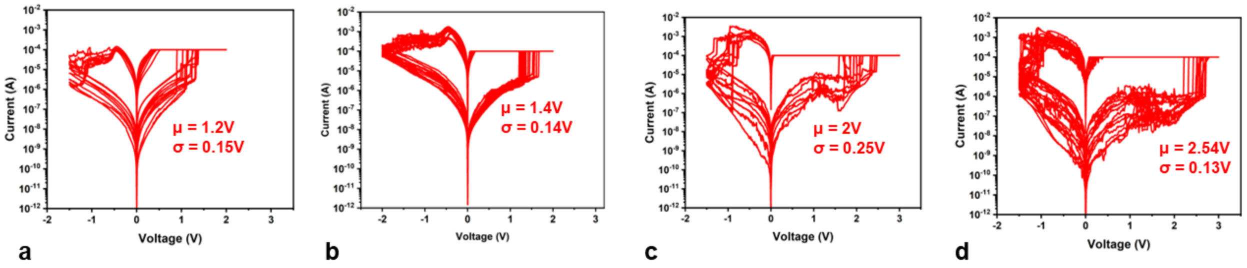

Supplementary figure 10: (a-d) DC IV characteristics measured from four different memristor devices.

We speculate that the electrode surface roughness contributes to the higher device-to-device variation among these devices. Evaporated metal electrodes typically have a surface roughness of about 1-2nm [S16-S17]. The effect of this roughness on the switching characteristics should be lower in memristors with thicker switching layer (>5nm). However, as the switching layer thickness scales down, it is easier for filaments to propagate vertically and form around weak spots with bumps/hillocks. Therefore, the surface roughness of the electrodes will largely determine the location and voltage of the breakdown. These variations can be reduced with industry-standard tools that can achieve lower surface roughness, which is difficult to achieve with university equipment.

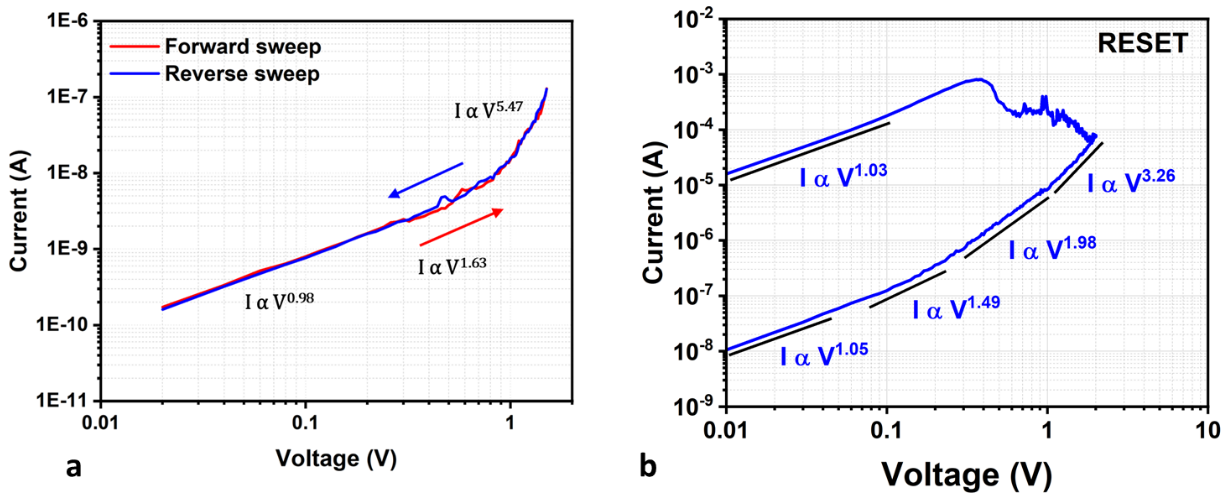

Supplementary figure 11: (a) DC current voltage sweep of a device in HRS. The reverse sweep (blue) traces the forward sweep (red) over the entire I-V range, indicating that the change in slope is caused by the transport mechanism inside the dielectric rather than the filament formation. (b) RESET I-V curve plotted on the log-log scale. The device exhibits linear conduction in the LRS and SCLC in the HRS.

Conductive Atomic Force Microscopy

To confirm the proposed filamentary conduction mechanism, we performed Conductive AFM (CAFM) measurements on our devices. Sample preparation process flow and experimental setup are shown in the schematic below (Supplementary Figure 12.1). This approach of using ionic liquid for CAFM measurements was adopted from previous work [S18].

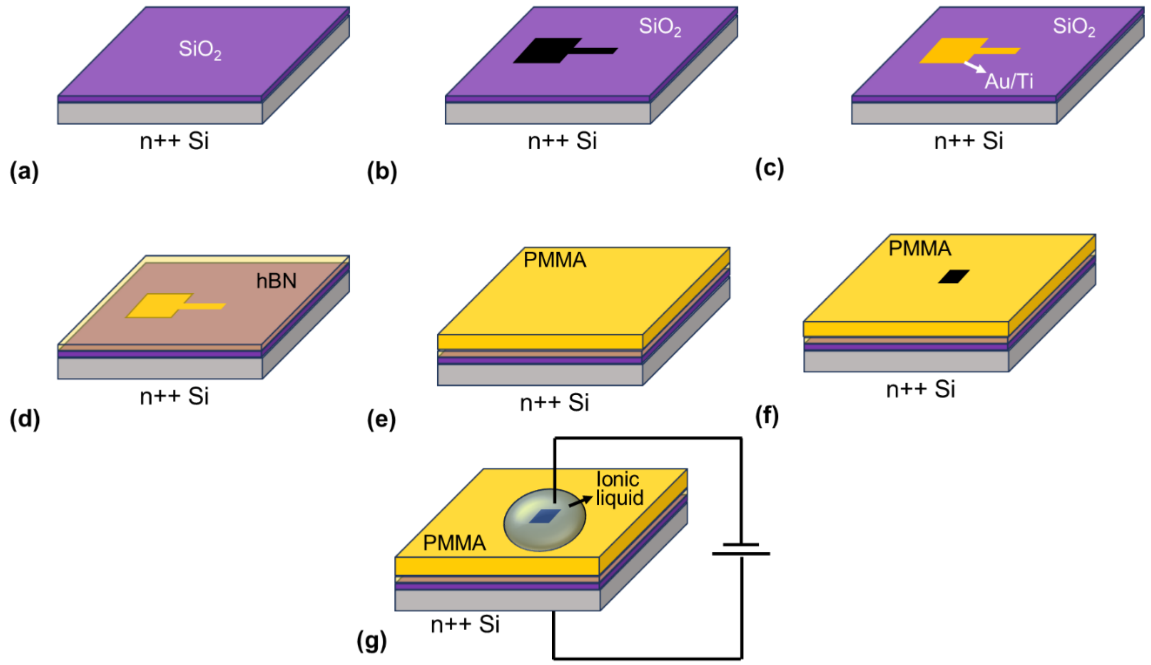

Supplementary Figure 12.1: Schematic showing the sample preparation process flow for Conductive AFM measurement. (a) Initial Si/SiO_2 substrate (SiO_2 thickness – 20nm) (b) Pattern and etch SiO_2 such that the Si surface is exposed (c) PVD deposition of Au followed by Ti (d) Transfer hBN film onto the bottom electrode (Ti comes in contact with hBN) (e) Spin coat PMMA layer (f) pattern and develop PMMA layer (small region 3x3um^2 exposed) (g) Drop ionic liquid (makes contact with hBN layer) and apply voltage bias.

The sample was prepared such that the titanium metal made contact with the hBN layer to emulate our device structure. Filaments were formed in the hBN layer by stressing the device as shown in Supplementary Figure 12.1(g) (positive voltage to the bottom electrode). After stressing the devices, the ionic liquid was washed away, PMMA was dissolved in acetone and then the samples were taken to CAFM for measurements. During the CAFM measurements, the AFM tip comes in contact with the hBN layer and the back-side silicon substrate is grounded. Large area scan (300nmx300nm) clearly shows the formation of conductive filaments (Supplementary Figure 12.2(a)) across the hBN layer. High resolution scan of a single conducting filament is presented in Supplementary Figure 12.2(b). Based on the CAFM and EELS measurements (Fig.2g, main text), we can conclude that the dominant switching mechanism in our devices originates from the formation/dissolution of Ti ion constituted conductive filaments.

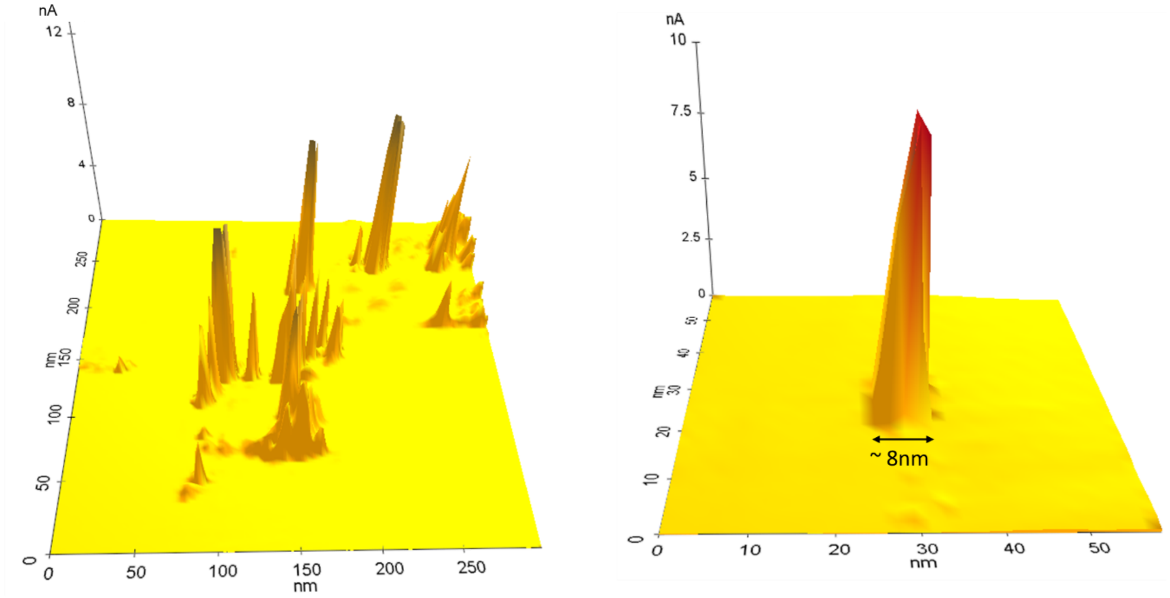

(a) **(b)**

Supplementary Figure 12.2: (a) Large area CAFM current map showing multiple filaments (b) High resolution current map of a single filament.

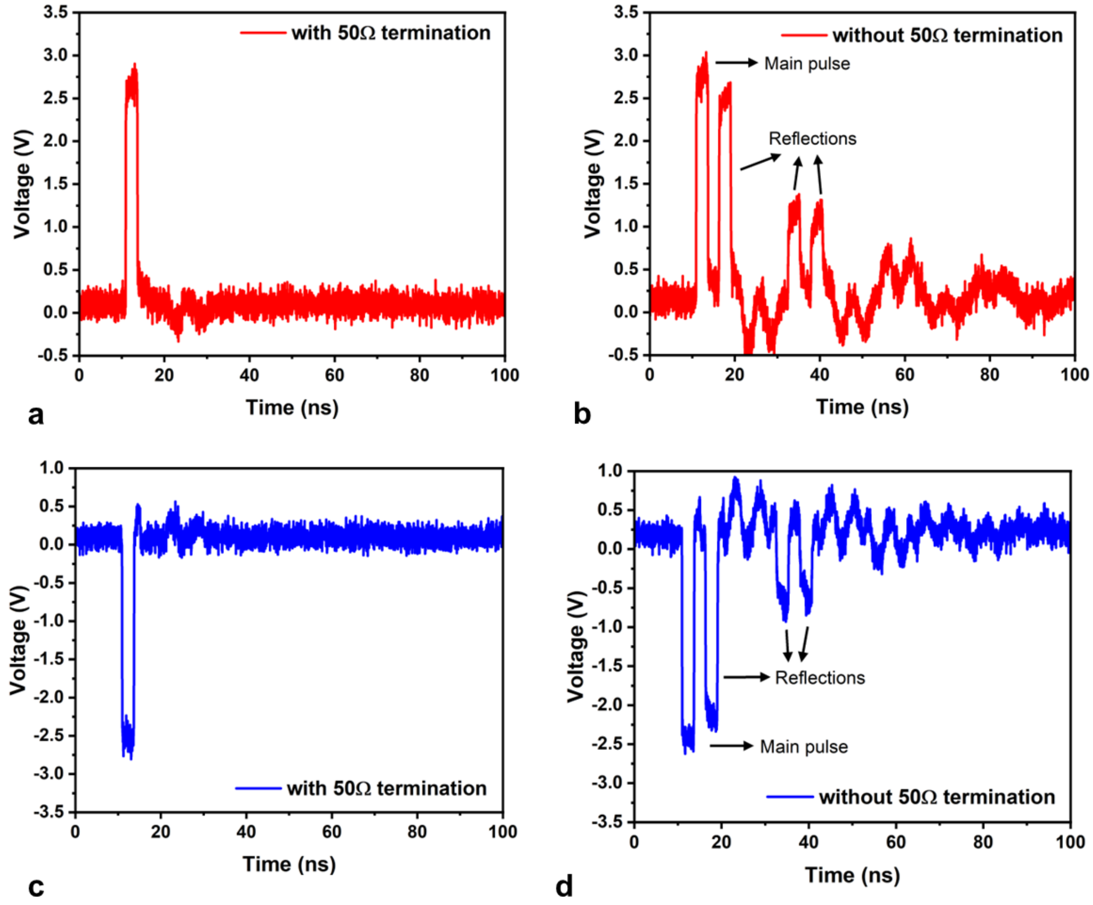

Supplementary figure 13: Transient waveform for SET pulse (a) with 50Ω termination (b) without 50Ω termination soldered onto the probe tips. RESET pulse (c) with 50Ω termination (d) without 50Ω termination soldered onto the probe tips. The 50Ω termination resistor effectively suppresses reflections and ensures maximum power transfer. Such reflections would result in random programming of the memristor. Therefore, all the experiments presented in this work were conducted with the 50Ω termination on the probe tips ensuring minimal reflected power.

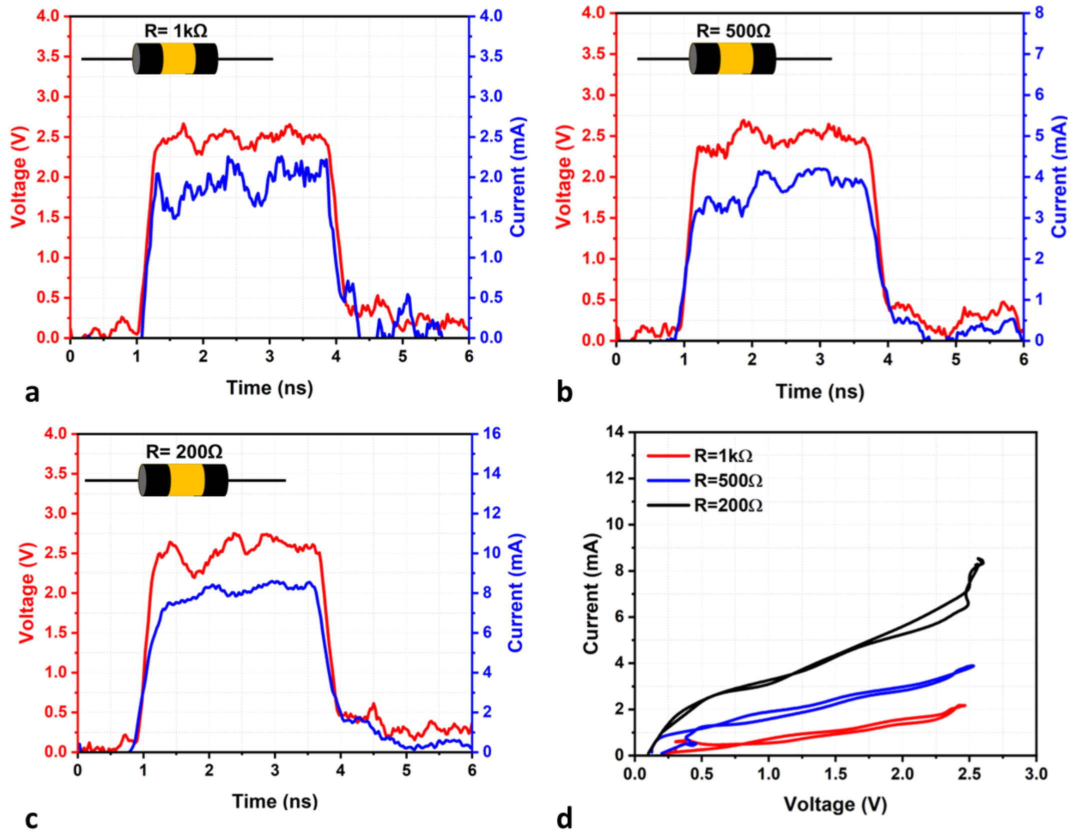

Supplementary figure 14: Applied voltage pulse (red trace) and measured current (blue trace) across a discrete surface mount resistor with the resistance (a) 1kΩ (red trace) (b) 500Ω (blue trace) (c) 200Ω (black trace). (d) I-V plots of the pulses are shown in (a),(b),(c). The graphs display no significant hysteretic window suggesting that the hysteresis in Fig. 3(c),(e) in the main text can be attributed to the resistive switching of the memristor rather than to setup parasitics.

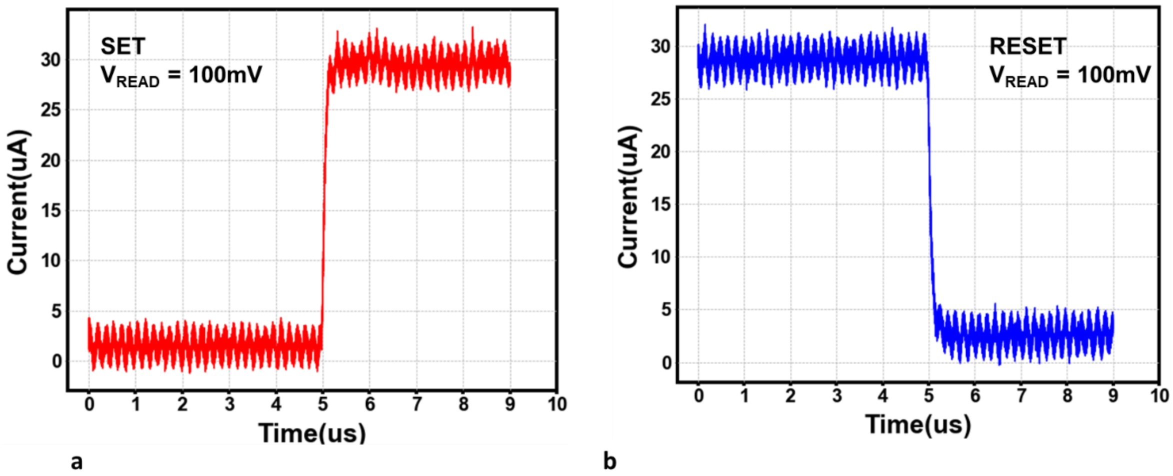

Supplementary Figure 15: (a) Current signal measured on the DC path during a SET operation with a background DC offset of 100mV. Change in resistance is evident, confirming the resistive switching in the memristor device (b) Current signal measured during a RESET operation.

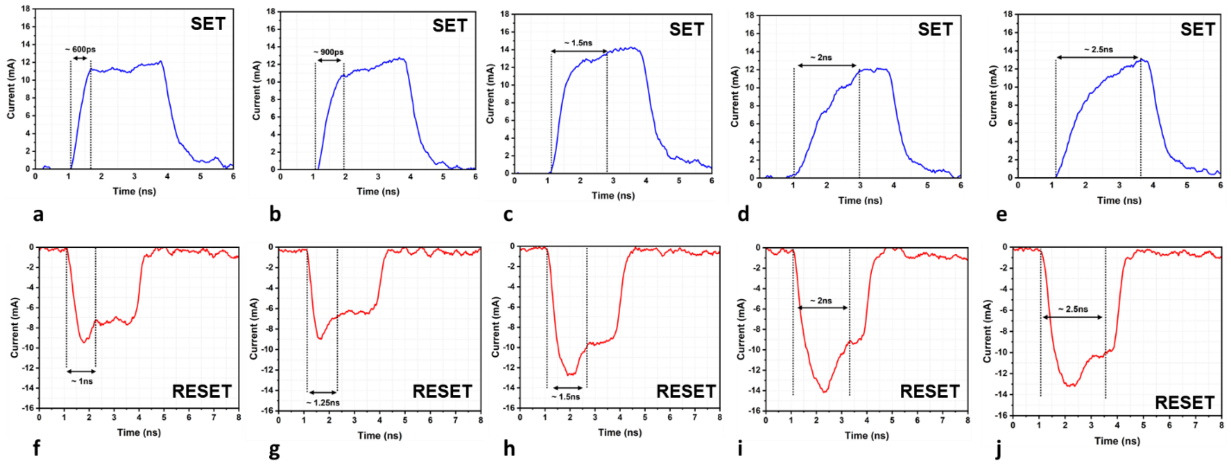

Supplementary Figure 16: SET current waveforms with intrinsic switching time (a) 600ps (b) 900ps (c) 1.5ns (d) 2ns (e) 2.5ns. The applied voltage signal is not included for clarity. RESET current waveforms with intrinsic switching time (f) 1ns (g) 1.25ns (h) 1.5ns (i) 2ns (j) 2.5ns.

Here the switching time is defined as the time taken for the current signal to settle to ~90% of its final magnitude. Most other studies do not include the rise time of the voltage pulse, and the switching time is usually reported as the time required to reach 50% of the final current magnitude. Though such characterization provides an attractive switching time estimate (results in T_{SWITCH} < 1ns in our measurement), we believe that such an approach does not reflect a realistic scenario. Therefore, the switching time data provided in this study should be taken as a realistic estimate. Data extracted from 200 different traces have been used for further analysis in Figure 4,5 in the main text.

Ultra-fast switching in 2D memristors

The switching mechanism in our devices is based on metal ion diffusion into the switching layer. The applied electric field facilitates the vertical propagation of the filament around weak spots in the dielectric. This initially formed narrow filament connects the electrodes and lowers the resistance of the memristor. The current flowing through this filament generates temperature through the Joule heating phenomenon. The temperature generated in the filament lowers the energy barrier for ion release from the electrode and further propels the filament growth. To understand the fundamental reason for the ultra-fast switching in our devices, we perform transient thermal analysis using finite element based physics solvers (COMSOL). To summarize the results, the characteristic properties aiding the ultra-fast switching in 2D hBN memristors are:

1. Ultra-thin switching layer
2. High thermal conductivity of 2D hBN layers

<u>Ultra-thin switching layer</u>

Oxide memristors (commercial or university demonstrations) typically have a thicker switching layer (5nm-20nm) in comparison to the memristors in this study (< 2.4nm). The thinner switching layer significantly increases the local electric field across the dielectric, which promotes faster resistance switching. Supplementary Fig.17.1a shows the electric field increases inside the switching layer as the dielectric thickness reduces.

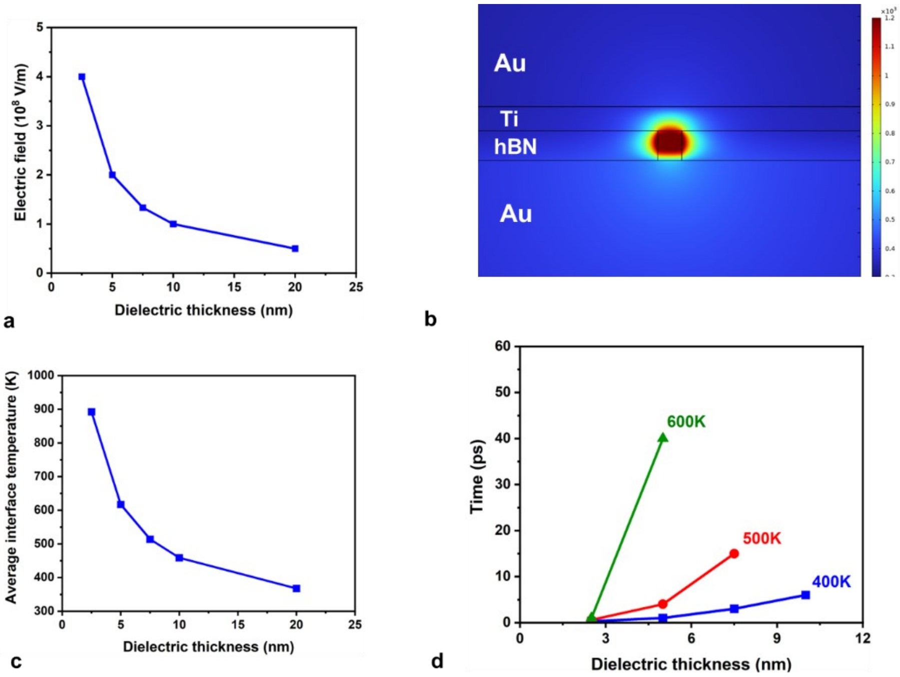

Supplementary Figure 17.1: (a) Electric field vs dielectric thickness for V=1V. (b) Snapshot of the COMSOL model showing the metallic filament and electrodes. The temperature profile shows the highest temperature at the center of the filament. (c) Temperature reaching the interface vs dielectric thickness (d) time taken for the interface to reach 400K (blue curve), 500K (red curve), 600K (green curve) as a function of dielectric thickness.

From the Arrhenius model [S19-S21], the filament growth dynamics depend on the temperature in addition to the applied voltage. The critical physical process that determines the switching speed of the device is the metal ion release [S19] which is determined by the Ti/hBN interface temperature (T_{INT}). Note that this temperature is different from the filament temperature (T_{CF}). The current passing through the device generates temperature in the filament (T_{CF}) through joule heating. This temperature is highest at the center of the filament (vertically) and lowers towards the electrodes, as seen in Supplementary Fig.17.1b. This type of temperature profile has been reported in several previous studies [S20-S22]. Most studies approximate $T_{CF} \sim T_{INT}$ for simplicity in analytical modeling.

Using the COMSOL model, we study the T_{INT} dependence on dielectric thickness, as shown in Supplementary Fig.17.1c. It is evident that as the dielectric thickness reduces, steady-state T_{INT} increases. In addition, Supplementary Fig.17.1d plots the time taken for temperature build-up at the interface as a function of dielectric thickness. We plot the time taken to reach 400K, 500K, and 600K for comparison. The T_{INIT} for thinner dielectric (such as the devices in this study) reaches 600K in less than 1ps, whereas thicker dielectric ~5nm takes about 40ps. The faster temperature build-up at the interface promotes faster metal ion release, which creates a positive feedback loop: higher T_{INT} → lower R → higher T_{INT}. This positive feedback loop promotes faster switching in devices with a thinner dielectric layer.

High thermal conductivity of 2D hBN

Based on the previous discussion, the obvious subsequent question would be - "Will the oxide RRAMs with thinner switching layer switch as fast as the 2D memristors?" To answer this question, we simulated two devices – one with an HfO_x layer and the other with 2D hBN, with identical switching layer thicknesses (2.5nm).

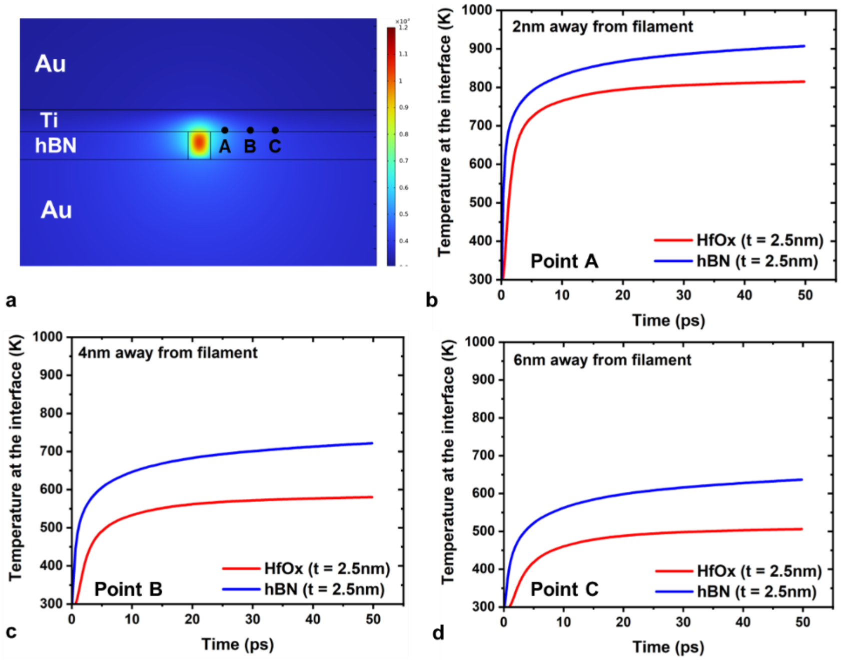

Supplementary Fig.17.2: (a) Snapshot of COMSOL model showing three points of interest A,B,C. Transient interface temperature at (b) point A (c) point B (d) point C for HfOx (red curve) and hBN (blue curve).

In addition to thickness, another physical property that determines the switching speed of the devices is the thermal conductivity of the switching layer. The in-plane thermal conductivity of hBN (k ~ 100-200 W/mK) [S23-S25] is significantly higher than oxides (k ~ 0.5-2 W/mK) typically used for memristors (HfO_2, Al_2O_3, TaO_x) [S26-S28]. On the other hand, the out-of-plane thermal conductivity is comparable (~5 mW/K) to the oxides. The high thermal conductivity quickly spreads the heat from the filament to the surroundings, which heats a larger cross-section of the interface. Supplementary Fig.17.2a shows three points on the interface at 2nm,4nm, and 6nm away from the filament. Temperature transients at these points (Supplementary Fig.17.2 b,c,d) highlight this effect. Evidently, T_{INT} increases rapidly for hBN compared to HfO_x. This effect is especially more pronounced as we move away from the filament (points B, C). Since a larger cross-section of the interface is heated, the probability of ion release increases, which results in faster filament expansion. Therefore, 2D materials that typically have higher thermal conductivity promote faster switching compared to oxide RRAMs with identical layer thickness.

Overall, the 2D memristors benefit from having an ultra-thin switching layer as well as high thermal conductivity compared to TMO memristors. Fig.4d (main text) shows the T_{INT} for hBN devices with 2.5nm thickness and HfOx devices with 5nm thickness (typical thickness for TMO memristors). Clearly, the temperature rises rapidly for the hBN devices, resulting in ultra-fast switching.

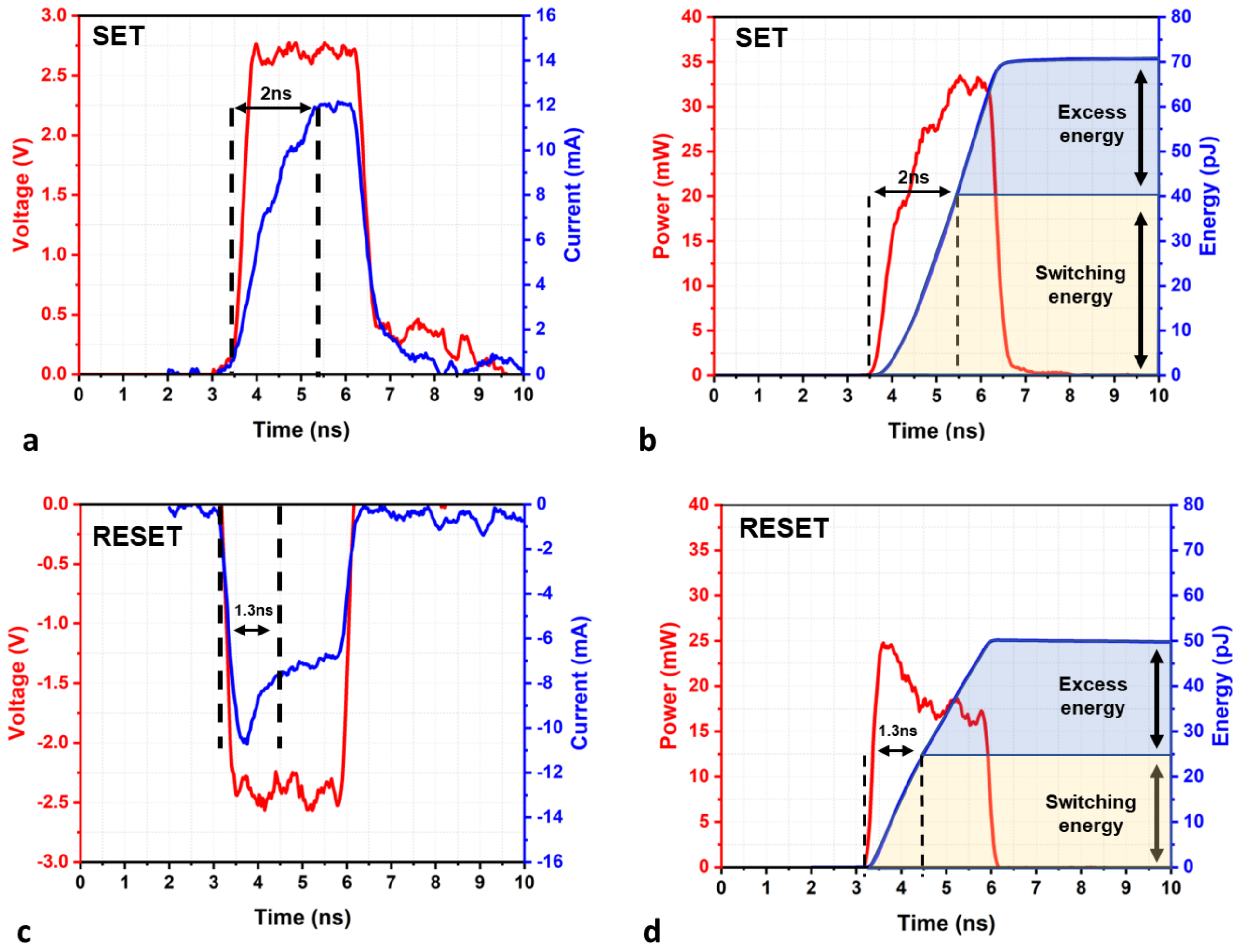

Supplementary figure 18: (a) Applied voltage pulse (red trace) and measured current (blue trace) in a SET transition. The device takes about 2ns to complete the transition to the final state (b) Power ($P = V * I$) consumed during the SET transition (red trace). The total energy obtained by integrating power over time ($E = \int P\, dt$) is shown in the blue trace. The total energy is partitioned into switching energy (energy contributed to switching) and excess energy (energy spent after the switching is complete), as discussed in the main text. (c) Applied voltage pulse (red trace) and measured current pulse (blue trace) for a RESET transition (d) Power and energy plots during the RESET transition.

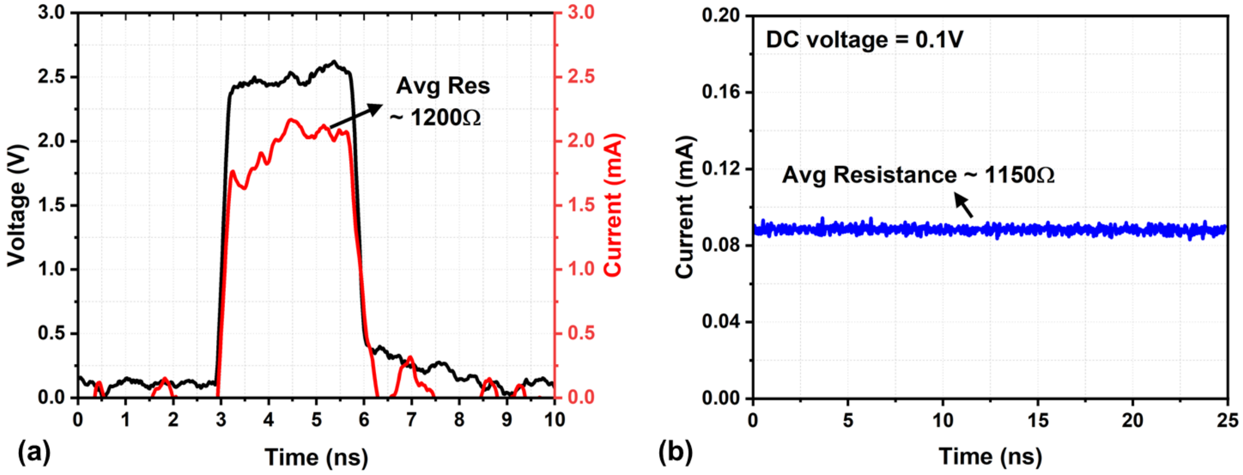

Supplementary figure 19: (a) Transient voltage (black trace) and current waveforms (red trace) applied to a discrete resistor (b) DC current measured before and after the pulse for the discrete resistor.

As explained in the methods section, the resistance during the pulse is directly measured by the oscilloscope, whereas the resistance before/after the pulse is measured through a current amplifier in the branched DC path. Therefore, any additional resistance in the DC path can increase the resistance after the pulse. Supplementary Fig.19 shows that the resistance during and after the pulse measured close to 1.2kΩ. Therefore, the resistance increase observed after the pulse in Fig.5c (main text) is indeed a characteristic feature of the memristors associated with Joule heating.

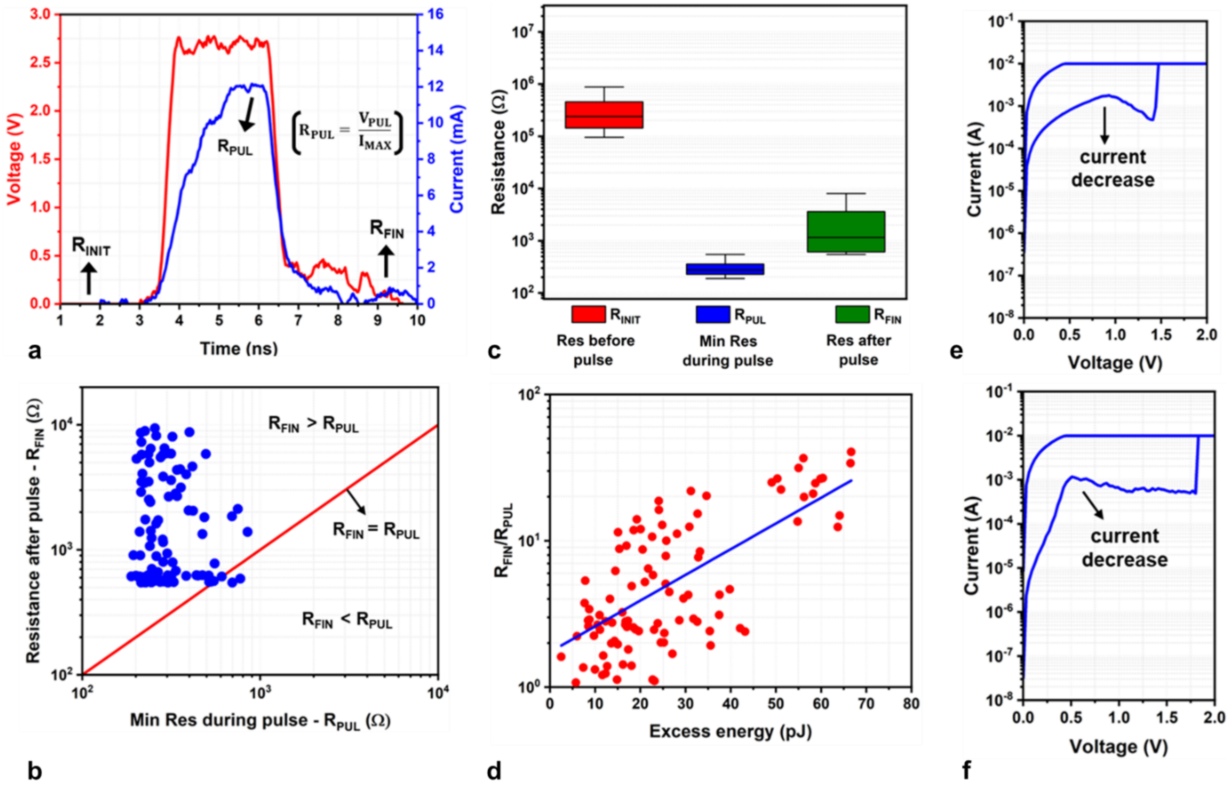

Supplementary Figure 20: (a) Voltage (red trace) and current pulse (blue trace) waveforms during a SET operation. The resistance value is calculated at the maximum current magnitude during the pulse (R_{PUL} - blue boxplot) and immediately before (R_{INIT} – red boxplot) and after (R_{FIN} – green boxplot) the pulse via applying small DC bias V=100mV (b) R_{FIN} vs. R_{PUL} collected from 100 transient traces in (a): The post-switching resistance R_{FIN} > R_{PUL} in ~97% of the collected data points (c) Resistance distribution of R_{INIT}, R_{PUL}, R_{FIN}. The distribution clearly indicates the filament resistance increases after the pulse voltage is scaled down. This resistance increase should not be confused with the resistance relaxation observed in volatile resistive switching devices since the currents in this study (~6mA-12mA) are higher than the volatile switching devices current limits (<100uA) [S18,S29]. Therefore, the data suggest that the filament cross-section is reduced. (d) The post-switching resistance increase (R_{FIN}/R_{PUL}) correlates (weakly) to a higher excess switching energy, implying that high temperature may induce random ions migration away from the filament, which suppresses the filament conductivity (e), (f) DC I-V sweep showing current decrease during a SET pulse. These rare instances of current decrease occur when RESET does not sufficiently dissolve the filament, and a large current promotes its further thermal dissolution that is consistent with the suggestion in (d). Such Joule heating induced resistance increase in 2D hBN memristors has been previously reported [S30].

Supplementary figure 21: Filament growth model

The switching mechanism in our devices is based on titanium ion migration into the hBN layer that form filaments. The filament growth model based on Arrhenius relationship is a universally accepted phenomenological model that satisfactorily captures the growth dynamics in TMO memristors and CBRAMs (conductive bridge RAM) [S19, S31-S33]. Therefore, in this study, we employ the same model with modifications to capture the behavior of our 2D memristors under ultra-fast voltage pulse stresses. Based on this model, the time dependency of the filament growth can be described by,

$$\frac{d\Phi}{dt} = A_1\, e^{\left(-\frac{Ea0-\alpha qV}{kT_{CF}}\right)} \qquad \text{---------- (1)}$$

where Φ → filament diameter

- A_1 → pre-exponent factor
- E_{a0} → Activation energy for bond breaking and ion hopping
- α → barrier lowering coefficient
- q → charge of electron
- V → voltage across the device
- k → Boltzmann's constant
- T_{CF} → conductive filament temperature

According to Eq.(1), the filament growth rate depends on the applied voltage as well as the temperature of the memristor device. Based on the equations presented in [S32], the thermal time constant of the filament can be calculated to be 0.226ps for our devices. As the thermal time constant is ~500x smaller than the input pulses (T_{PULSE} ~ 120ps-3ns), any transient effects can be safely neglected. Therefore, the conductive filament temperature can be obtained by solving the steady-state Fourier heat equation.

The ramping voltage pulse facilitates the vertical propagation of the filament around weak spots in the dielectric. This initially formed narrow filament connects the electrodes and lowers the resistance, which increases the current in the memristor. The local temperature (T_{CF}), determined by Joule heating of the filament ($T_{CF} \propto V*I$), in turn, increases with current that further propels the filament growth. This self-accelerating process – larger Φ → larger I → larger T → larger Φ proceeds until the device enters a high current regime where secondary effects begin to manifest. In the high current regime (I>5mA), the external resistance R_C (arising from contact leads) results in a voltage drop, thereby lowering the effective voltage across the device (V_{DEV}). In addition, T_{CF} increases significantly in the high current regime, which increases the out-diffusion of ions from the filament, thus promoting the dissolution of the filament. The out-diffusion of ions from the filament can be modeled by,

$$\frac{d\Phi}{dt} = -A_2 e^{\left(-\frac{Ea}{kT_{CF}}\right)} \qquad \text{---------- (2)}$$

where Ea is an activation energy for out-diffusion of ions from the filament. The overall filament growth rate can be modeled by combining Eq.(1) and Eq.(2),

$$\frac{d\Phi}{dt} = A_1 e^{\left(-\frac{Ea0-\alpha qV}{kT_{CF}}\right)} - A_2 e^{\left(-\frac{Ea}{kT_{CF}}\right)} \quad \text{--------- (3)}$$

The first term in Eq.(3) represents the filament growth due to ion diffusion from the Ti electrode and the second term describes the filament dissolution due to ion out-diffusion from the filament. During the switching energy phase, when the Joule heating is insignificant (low current in the memristor), the filament growth rate dominates, resulting in filament expansion. On the other hand, during the excess energy phase, when Joule heating is significant (high current in the memristor), the filament dissolution rate increases. Here, a stable equilibrium is established between filament growth and dissolution processes. The system restores back to the equilibrium state when disturbed. For example, let us assume that the second term in Eq.(3) begins to dominate, then the following events occur: Φ decreases → R increases → T_{CF} decreases → Φ increases. This negative feedback loop ensures that the filament diameter and the corresponding current through the device saturate after reaching a certain maximum, as evident from Fig.3b in the main text. Finally, during the voltage pulse falling transition, the filament growth rate (determined by V, T_{CF}) recedes faster than the filament dissolution rate (determined only by T_{CF}), thereby resulting in the narrowing of the filament.

The time evolution of the filament can be calculated by self-consistently solving for conductive filament diameter (Φ) and temperature (T_{CF}) as shown by the flowchart in Supplementary Fig.21.1,

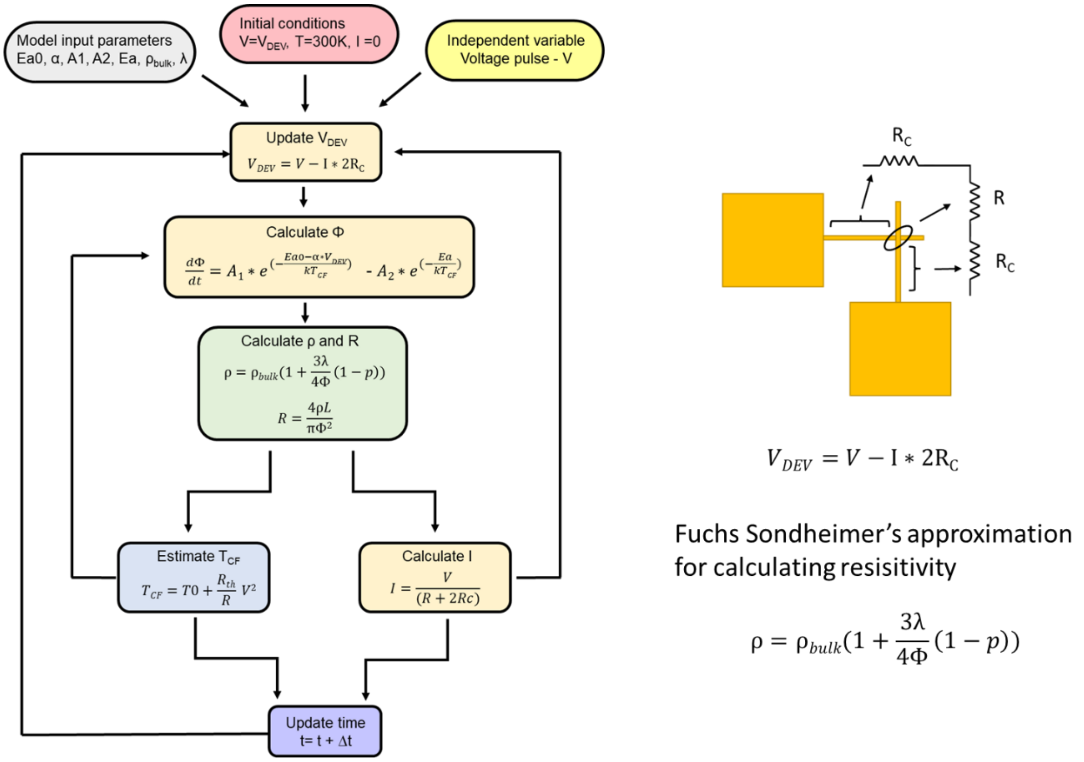

Supplementary Figure 21.1: Flowchart highlighting the sequence of equations providing the self-consistent solution.

The input parameters included in the model are listed in the table below. The transient voltage pulse is an independent variable to the model. The model iterates over time while calculating and updating Φ, R, T_{CF}, V_{DEV} over each iteration using the following equations,

- Eq.3 for calculating Φ
- Fuchs-Sondheimer's approximation [S34] for calculating dimension dependent resistivity and corresponding resistance
- Fourier's steady-state equation for calculating T_{CF}
- Ohm's law to update V_{DEV}

Parameter	Value	Parameter	Value
E_{a0}	1.2eV [S32]	ρ_{bulk}	42e-8 Ωm [S34]
α	0.4 [S32]	λ	28nm [S35]
A_1	1nm/s-10nm/s [S32]	p	0.5 [S35]
E_a	0.3eV	T_0	300K
A_2	1nm/s – 10nm/s[S19]	R_C	20Ω-80Ω

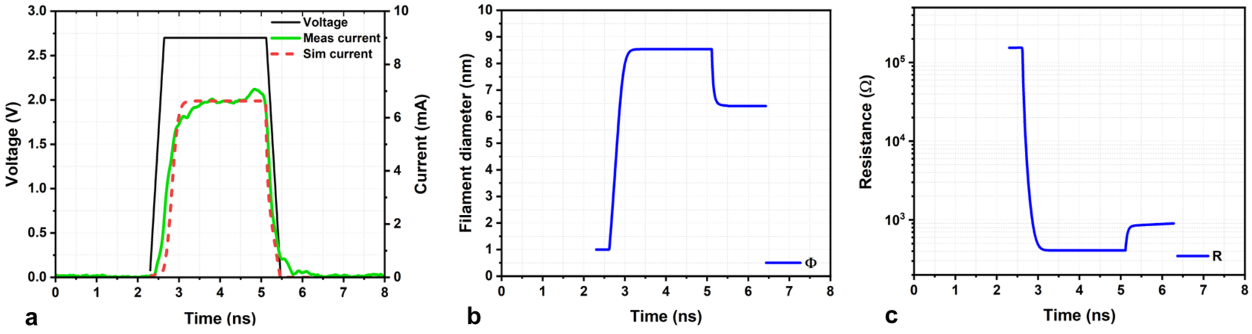

Supplementary Figure S21.2: SET Calculated time evolution of (a) I (b) Φ (c) R for a device with T_{SWITCH}=700ps (fig.3b in the main text). The model current (red trace) closely fits the measured current waveform (green trace) for an applied voltage pulse (black trace).

The self-consistent electro-thermal solver using the proposed model (red dotted trace) closely fits the measured data (green solid trace) (Supplementary Fig.21.2). In addition, the model also captures the filament narrowing. The filament expands to ~9nm in diameter and narrows to ~6.5nm after pulse termination. The memristor resistance change closely matches the resistance values observed in Fig.5c main text and Supplementary Fig.20. We believe that this joule heating driven filament narrowing will be observed in other 2D memristors as well, and therefore the proposed equations can be used to model them.

Similarly, the filament dynamics during RESET can be captured using Eq.4,

$$\frac{d\Phi}{dt} = -A_1 e^{\left(-\frac{Ea0-\alpha qV}{kT_{CF}}\right)} - A_2 e^{\left(-\frac{Ea}{kT_{CF}}\right)} \qquad \text{---------} \ (4)$$

Here, the term associated with Joule heating accelerates filament dissolution and aids the RESET process. The electro-thermal solver using the above equation closely models the measured RESET current signature as seen from Fig.S20.3a.

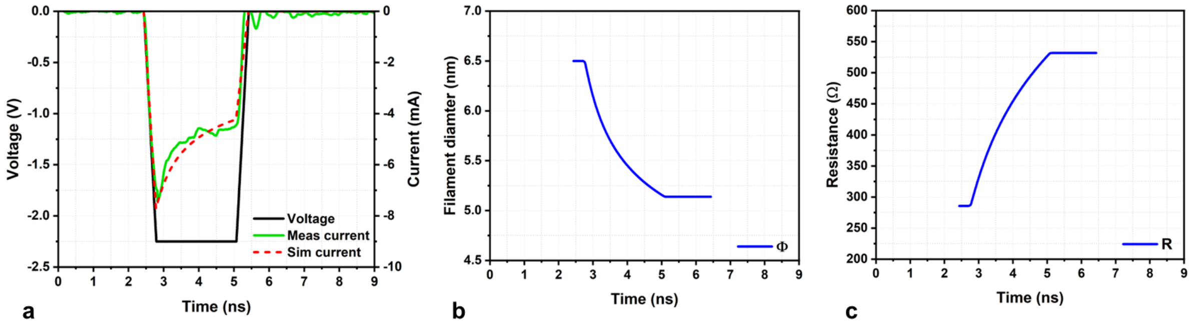

Supplementary Figure S21.3: RESET Calculated time evolution of (a) I (b) Φ (c) R for a device with T_{SWITCH}=1ns (fig.3d in main text). The model current (red trace) closely fits the measured current waveform (green trace) for an applied voltage pulse (black trace).

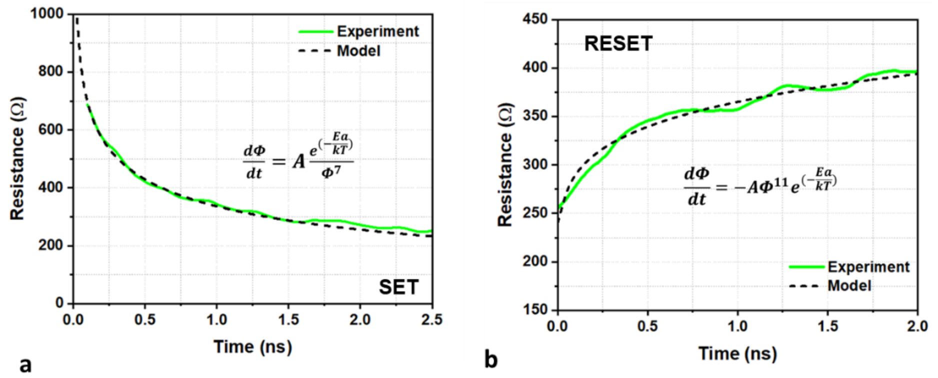

Supplementary Figure 22: (a) Resistance time dependency during SET operation (b) Resistance time dependency during RESET operation. The model (black dotted line) closely fits the experimental data (green solid line).

<u>SET operation – Empirical model</u>

The filament growth is modeled using the Arrhenius equation modified by including filament enlargement factor (Φ^n) to account for growth rate deceleration process. This simple empirical model of resistance evolution over time assumes a constant temperature and, therefore, significantly reduces the computational time. Such models can be employed to study large array level performance metrics using SPICE circuit simulations. Supplementary Fig.22a shows the measured data (solid green trace) and simulations (black dotted trace) for the resistance time dependency: the growth rate of the filament diameter is accurately reproduced. The following equations are used in this model:

$$\frac{d\Phi}{dt} = \frac{A * exp\left(-\frac{Ea}{kT}\right)}{\Phi^n}$$

$$\Phi = (n+1)\ (A * \exp\left(-\frac{Ea}{kT}\right) * t)^{1/(n+1)}$$

$$R = \frac{4 * \rho * L}{\pi(n+1)^2\ (\ A * \exp\left(-\frac{Ea}{kT}\right) * t)^{2/(n+1)}}$$

<u>RESET operation – Empirical model</u>

Like SET, the RESET operation also displays filament diameter dependent dissolution rate: the R(t) slope decreases as the filament dissolves. Supplementary Fig.22b shows the measured data (solid green trace) and simulations (black dotted trace) for the resistance time dependency: the model closely matches the measured data. The following equation are used in the model,

$$\frac{d\Phi}{dt} = -\Phi^n * A * exp(-\frac{Ea}{kT})$$

$$\Phi = \frac{1}{(n-1) * A * exp\left(-\frac{Ea}{kT}\right) * t + ((n-1) * \Phi_0)^{1-n}}$$

$$R = \frac{4*\rho*L}{\pi}((n-1)*A*exp\left(-\frac{Ea}{kT}\right)*t+((n-1)*\Phi_0)^{1-n})^{2/(n-1)} *$$

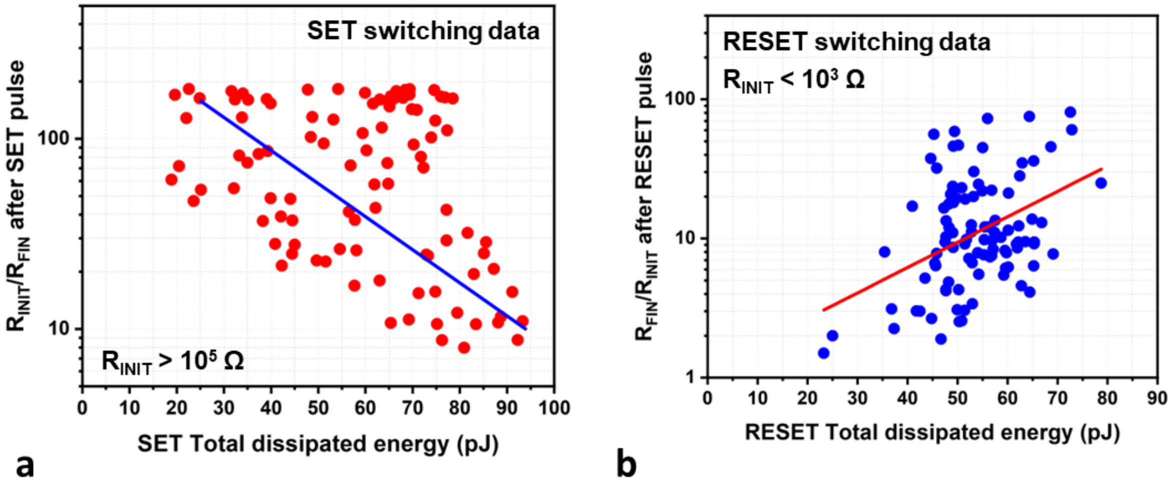

Supplementary Figure 23: (a) Relative resistance decrease after SET, (R_{INIT}/R_{FIN}) vs total energy (switching energy + excess energy). A downward trend indicates that the greater total energy results in smaller resistance decrease induced by a SET pulse. Higher energy dissipation (higher Joule heating) can suppress the filament by assisting with post-switching out-diffusion of metal ions, as discussed in Supplementary Fig. 20 (b) Relative resistance increase after the RESET pulse (R_{FIN}/R_{INIT}) vs total energy (switching energy + excess energy). An upward trend indicates that higher dissipation aids the RESET process resulting in larger resistance increase.

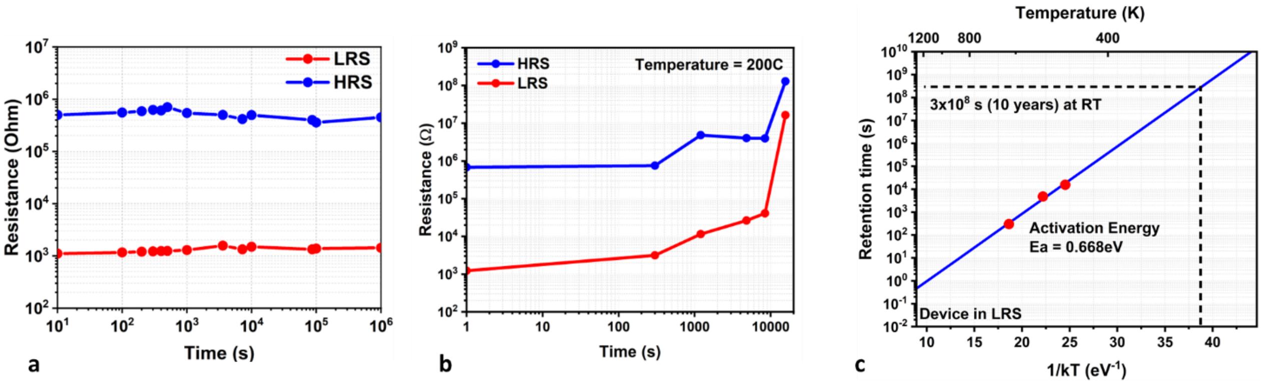

Supplementary Figure 24: (a) Retention measurement at room temperature. Devices retain their state with minimal drift in characteristics up to 10^6 seconds (11 days). The measurement was discontinued after 11 days. (b) Memristor retention at 200C. Resistance increases over time (both in LRS (red curve) and HRS (blue curve)) confirming the role of heat in filament dissolution (c) LRS retention time at different operating temperatures. The LRS retention time shows an exponential dependence on the temperature, characterized by an activation energy of 0.668eV. Extrapolation shows that the device could retain the state for 10 years at room temperature.

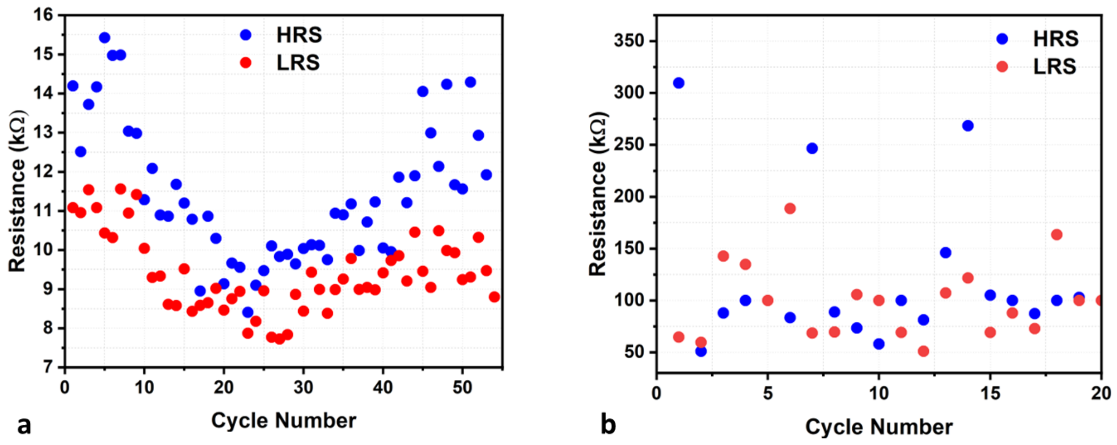

Supplementary Figure 25: (a)(b) Endurance cycling with ultra-short pulses (T_{PULSE} ~ 120ps) for thick hBN film (~ 7-8 layers). HRS data points shown in blue and LRS data points shown in red. Switching is inconsistent, no clear memory window is observed contrary to thin hBN film devices (Fig. 6c in main text).

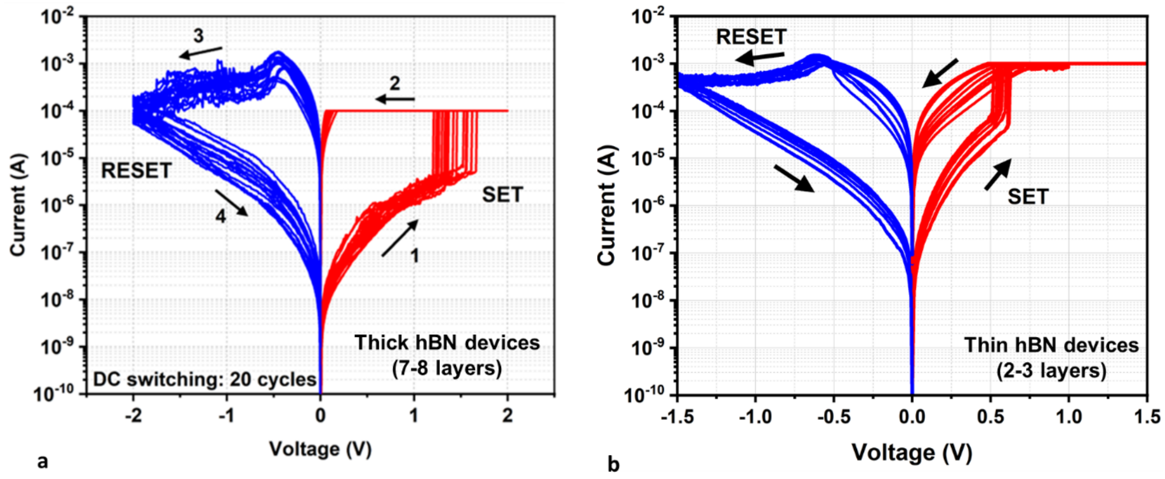

Supplementary Figure 26: (a) Representative DC I-V characteristics of a thick hBN device (7-8 layers) and (b) a thin hBN device (3-4 layers). SET curves shown in red and RESET curves shown in blue. Thin hBN devices do not require a forming process to establish the initial conductive filament and have a smaller memory window compared to thick devices.

.

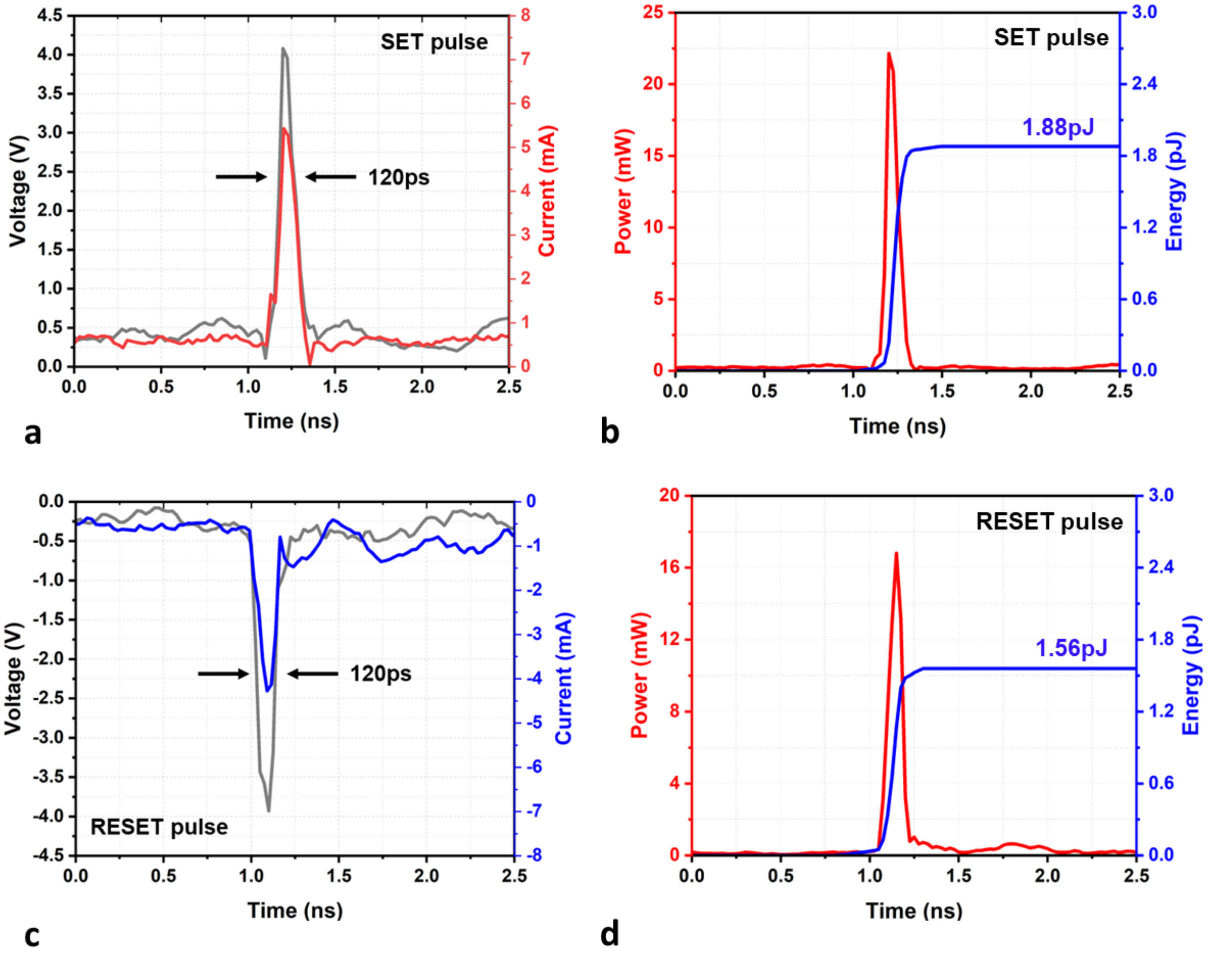

Supplementary Figure 27: (a) Ultra-short voltage pulse (grey trace) and measured current (red trace) during SET transition. The resolution of the measurement setup (~25ps) allows only up to 4-5 data-points to be captured during the pulse stress that limits our ability to identify transient current features (similar to nanosecond pulses in Figures 3,4,5 in the main text). The switching is confirmed by the DC read operation after the pulse (b) Instantaneous Power (red trace) and energy dissipated (blue trace) during SET transition. The energy of < 2pJ is among the lowest reported switching energy in 2D material memristors (c) Ultra-short voltage pulse (grey trace) and measured current (blue trace) during the RESET transition (d) Power and energy dissipated during the RESET transition.

		Material	Switching time	Number of cycles presented	Reference
1		Yitrium Iron Garnet	540ps	20	S36
2		HfO_x	400ps	1	S37
3	Transition Metal Oxide	HfO_2	100ps	1	S38
4		AlN	85ps	1	S39
5		HfOx	300ps	1	S40
6		SiO_2	105ps	10	S41
7		TaO_x	100ps	1	S42
		TaO_x	250ps	1	S43
8		$MoTe_2$	5ns	1	S44
9		MoS_2	15ns	1	S45
10		MoS_2	700ps	1	S46
11	2D Material	MoS_2	15ns	1	S47
12		$MoTe_2$	100ns	1	S48
		hBN (this work)	1.43ns	600	-
13		hBN (this work)	120ps	100	-

Supplementary Table1 – Comparison of switching times with previously published reports. The table includes fastest reported switching data from well-studied TMO memristors as well as emerging 2D memristors. The devices reported in this study outperform 2D memristors in terms of both speed and endurance.

Although a few studies report higher endurance at 100ps switching speed [S49-S50], they employ program-verify scheme during endurance cycling. In this approach, the device is subjected to repeating pulses until the desired transition (SET and RESET) is completed and several cycles may involve multiple pulses for successful operation. For the evaluation consistency, such studies have been omitted from this analysis. Moreover, if the device requires multiple pulses (even on a few instances), the switching speed cannot be reliably characterized.

Endurance in 2D memristors

The endurance in 2D memristors has been a concern, mainly attributed to the ultra-thin switching layer. Transition metal oxide (TMO) based memristors typically have a switching layer thickness ranging between 5nm-20nm [S51-S53]. On the contrary, 2D memristors typically have ultra-thin switching layers, sometimes even scaled down to monolayer thickness. The reduced vertical separation between metal electrodes enhances the electric field, which results in weaker control over the metal ion migration into the switching layer and subsequent filament formation. Moreover, as the thickness of the switching layer reduces, the impact of bottom/top electrode roughness plays a significant role in determining the overall device performance. In the following table, we have listed the reported endurance in 2D memristors along with the thickness of the switching layer.

	Switching layer stack	Thickness (nm)	Cycles endurance
[S54]	Au/hBN/Au	0.33	50
[S55]	Au/MoS_2/Au	0.65	20
[S56]	Au/MoS_2/Au	0.65	150
[S57]	Ag/$hBNO_x$/Gr	0.9	100
[S58]	Au/$ReSe_2$/Au	1	200
[S59]	Cu/MoS_2/Au	1.4	20
[S60]	Ti/hBN/Au	2.4	100
[S61]	Ti/hBN/Au	2.4	100
[S62]	Ti/hBN/Ni	3.96	100
[S63]	Ti/hBN/Au	4.3	50
[S64]	Ti/hBN/Au	4.95	250
[S65]	Ti/hBN/Gr	5	1000
[S66]	Au/MoS_2/Au	5	120
[S67]	Au/hBN/Au	6	50
[S68]	Pd/WS_2/Pt	6.45	15
[S69]	Ti/hBN/Au	6.6	1200
[S44]	Ti/$MoTe_2$/Au	7	600
This work	Ti/hBN/Au	2.5	600
This work	Ti/hBN/Au	1.32	100

Supplementary Table 2 – Comparison of reported endurance in 2D memristors.

From the table, we see that the 2D memristors with switching layer thickness <5nm, typically exhibit <250 cycles endurance. Some memristors [S65,S69] exhibit about 1000 cycle endurance with >5nm switching layer thickness. Our devices exhibit on-par performance with 600 cycles for thicker (T_{hBN}~ 2.5nm) memristors and 100 cycles for thinner (T_{hBN}~ 1.32nm) memristors.

On the other hand, only two research studies report high endurance (~10^7) using thicker 2D memristors [S70-S71]. Many studies report low endurance even with thicker 2D memristors [S72-S74].

Role of Current Compliance (CC)

A recent study [S75] reported high endurance of ~10^6 cycles in the 1T1M (1transistor-1memristor) bitcell with just ~6.6nm thick switching layer. In that study, the standalone devices without CMOS transistors exhibited only 100 cycles, whereas devices with transistors exhibited a million cycles. This promising result suggests that the adjoining selector device can improve endurance in 2D memristor devices.

The characterization setup utilized in this study cannot support external current compliance (CC) for high-frequency pulse testing. For the frequency range studied in this article, the external CC device (resistor, diode or transistor) must be located within <1cm distance from the DUT (device under test) to avoid refelctions. Therefore, the only possible option is to have a CC device fabricated monolithically, like in [S75]. Without current compliance, most of our devices get shorted during testing, which cannot be recovered. We expect that having a 1T1M configuration would improve the endurance of our devices as well.

Supplementary References

[S1] Kim, K.K., Hsu, A., Jia, X., Kim, S.M., Shi, Y., Hofmann, M., Nezich, D., Rodriguez-Nieva, J.F., Dresselhaus, M., Palacios, T. and Kong, J., 2012. Synthesis of monolayer hexagonal boron nitride on Cu foil using chemical vapor deposition. *Nano letters*, *12*(1), pp.161-166.

[S2] Lee, K.H., Shin, H.J., Lee, J., Lee, I.Y., Kim, G.H., Choi, J.Y. and Kim, S.W., 2012. Large-scale synthesis of high-quality hexagonal boron nitride nanosheets for large-area graphene electronics. *Nano letters*, *12*(2), pp.714-718.

[S3] Koepke, J.C., Wood, J.D., Chen, Y., Schmucker, S.W., Liu, X., Chang, N.N., Nienhaus, L., Do, J.W., Carrion, E.A., Hewaparakrama, J. and Rangarajan, A., 2016. Role of pressure in the growth of hexagonal boron nitride thin films from ammonia-borane. *Chemistry of Materials*, *28*(12), pp.4169-4179.

[S4] Tay, R.Y., Griep, M.H., Mallick, G., Tsang, S.H., Singh, R.S., Tumlin, T., Teo, E.H.T. and Karna, S.P., 2014. Growth of large single-crystalline two-dimensional boron nitride hexagons on electropolished copper. *Nano letters*, *14*(2), pp.839-846.

[S5] Sridhara, K., Feigelson, B.N., Wollmershauser, J.A., Hite, J.K., Nath, A., Hernández, S.C., Fuhrer, M.S. and Gaskill, D.K., 2017. Electrochemically prepared polycrystalline copper surface for the growth of hexagonal boron nitride. *Crystal Growth & Design*, *17*(4), pp.1669-1678.

[S6] Li, X., Magnuson, C.W., Venugopal, A., Tromp, R.M., Hannon, J.B., Vogel, E.M., Colombo, L. and Ruoff, R.S., 2011. Large-area graphene single crystals grown by low-pressure chemical vapor deposition of methane on copper. *Journal of the American Chemical Society*, *133*(9), pp.2816-2819.

[S7] Chen, S., Ji, H., Chou, H., Li, Q., Li, H., Suk, J.W., Piner, R., Liao, L., Cai, W. and Ruoff, R.S., 2013. Millimeter-size single-crystal graphene by suppressing evaporative loss of Cu during low pressure chemical vapor deposition. *Advanced Materials*, *25*(14), pp.2062-2065.

[S8] Gao, L., Ren, W., Xu, H., Jin, L., Wang, Z., Ma, T., Ma, L.P., Zhang, Z., Fu, Q., Peng, L.M. and Bao, X., 2012. Repeated growth and bubbling transfer of graphene with millimetre-size single-crystal grains using platinum. *Nature communications*, *3*(1), pp.1-7.

[S9] De La Rosa, C.J.L., Sun, J., Lindvall, N., Cole, M.T., Nam, Y., Löffler, M., Olsson, E., Teo, K.B. and Yurgens, A., 2013. Frame assisted H2O electrolysis induced H2 bubbling transfer of large area graphene grown by chemical vapor deposition on Cu. *Applied Physics Letters*, *102*(2), p.022101.

[S10] Solozhenko, V.L., Will, G. and Elf, F., 1995. Isothermal compression of hexagonal graphite-like boron nitride up to 12 GPa. *Solid state communications*, *96*(1), pp.1-3.

[S11] Marini, A., García-González, P. and Rubio, A., 2006. First-principles description of correlation effects in layered materials. *Physical review letters*, *96*(13), p.136404.

[S12] Shi, Y., Hamsen, C., Jia, X., Kim, K.K., Reina, A., Hofmann, M., Hsu, A.L., Zhang, K., Li, H., Juang, Z.Y. and Dresselhaus, M.S., 2010. Synthesis of few-layer hexagonal boron nitride thin film by chemical vapor deposition. *Nano letters*, *10*(10), pp.4134-4139.

[S13] Kuzuba, T., Era, K., Ishii, T. and Sato, T., 1978. A low frequency Raman-active vibration of hexagonal boron nitride. *Solid state communications*, *25*(11), pp.863-865.

[S14] Lin, W.H., Brar, V.W., Jariwala, D., Sherrott, M.C., Tseng, W.S., Wu, C.I., Yeh, N.C. and Atwater, H.A., 2017. Atomic-scale structural and chemical characterization of hexagonal boron nitride layers synthesized at the wafer-scale with monolayer thickness control. *Chemistry of Materials*, *29*(11), pp.4700-4707.

[S15] NIST X-ray Photoelectron Spectroscopy Database, NIST Standard Reference Database Number 20, National Institute of Standards and Technology, Gaithersburg MD, 20899 (2000), doi:10.18434/T4T88K, (retrieved [date of access]).

[S16] Yang, Z., Liu, C., Gao, Y., Wang, J. and Yang, W., 2016. Influence of surface roughness on surface plasmon resonance phenomenon of gold film. *Chinese Optics Letters*, *14*(4), p.042401.

[S17] Stenzel, O., Wilbrandt, S., Stempfhuber, S., Gäbler, D. and Wolleb, S.J., 2019. Spectrophotometric characterization of thin copper and gold films prepared by electron beam evaporation: thickness dependence of the Drude damping parameter. *Coatings*, *9*(3), p.181.

[S18] Shi, Y., Liang, X., Yuan, B., Chen, V., Li, H., Hui, F., Yu, Z., Yuan, F., Pop, E., Wong, H.S.P. and Lanza, M., 2018. Electronic synapses made of layered two-dimensional materials. Nature Electronics, 1(8), pp.458-465.

[S19] Huang, P., Liu, X.Y., Chen, B., Li, H.T., Wang, Y.J., Deng, Y.X., Wei, K.L., Zeng, L., Gao, B., Du, G. and Zhang, X., 2013. A physics-based compact model of metal-oxide-based RRAM DC and AC operations. *IEEE transactions on electron devices*, *60*(12), pp.4090-4097.

[S20] Niraula, D. and Karpov, V.G., 2017. Heat transfer in filamentary RRAM devices. *IEEE Transactions on Electron Devices*, *64*(10), pp.4106-4113.

[S21] Ielmini, D., Nardi, F. and Cagli, C., 2011. Physical models of size-dependent nanofilament formation and rupture in NiO resistive switching memories. *Nanotechnology*, *22*(25), p.254022.

[S22] Larentis, S., Nardi, F., Balatti, S., Gilmer, D.C. and Ielmini, D., 2012. Resistive switching by voltage-driven ion migration in bipolar RRAM—Part II: Modeling. *IEEE Transactions on Electron Devices*, *59*(9), pp.2468-2475.

[S23] Jo, I., Pettes, M.T., Kim, J., Watanabe, K., Taniguchi, T., Yao, Z. and Shi, L., 2013. Thermal conductivity and phonon transport in suspended few-layer hexagonal boron nitride. *Nano letters*, *13*(2), pp.550-554.

[S24] Alam, M.T., Bresnehan, M.S., Robinson, J.A. and Haque, M.A., 2014. Thermal conductivity of ultra-thin chemical vapor deposited hexagonal boron nitride films. *Applied Physics Letters*, *104*(1).

[S25] Jana, M. and Singh, R.N., 2018. Progress in CVD synthesis of layered hexagonal boron nitride with tunable properties and their applications. *International Materials Reviews*, *63*(3), pp.162-203.

[S26] Panzer, M.A., Shandalov, M., Rowlette, J.A., Oshima, Y., Chen, Y.W., McIntyre, P.C. and Goodson, K.E., 2009. Thermal properties of ultrathin hafnium oxide gate dielectric films. *IEEE Electron Device Letters*, *30*(12), pp.1269-1271.

[S27] Cappella, A., Battaglia, J.L., Schick, V., Kusiak, A., Lamperti, A., Wiemer, C. and Hay, B., 2013. High Temperature Thermal Conductivity of Amorphous Al2 O 3 Thin Films

Grown by Low Temperature ALD. *Advanced Engineering Materials*, *15*(11), pp.1046-1050.

[S28] Landon, C.D., Wilke, R.H., Brumbach, M.T., Brennecka, G.L., Blea-Kirby, M., Ihlefeld, J.F., Marinella, M.J. and Beechem, T.E., 2015. Thermal transport in tantalum oxide films for memristive applications. *Applied Physics Letters*, *107*(2).

[S29] Shi, Y., Pan, C., Chen, V., Raghavan, N., Pey, K.L., Puglisi, F.M., Pop, E., Wong, H.S. and Lanza, M., 2017, December. Coexistence of volatile and non-volatile resistive switching in 2D h-BN based electronic synapses. In 2017 IEEE International Electron Devices Meeting (IEDM) (pp. 5-4). IEEE.

[S30] Zhuang, P., Ma, W., Liu, J., Cai, W. and Lin, W., 2021. Progressive RESET induced by Joule heating in hBN RRAMs. *Applied Physics Letters*, *118*(14), p.143101.

[S31] Yu, S. and Wong, H.S.P., 2011. Compact modeling of conducting-bridge random-access memory (CBRAM). *IEEE Transactions on Electron devices*, *58*(5), pp.1352-1360

[S32] Ielmini, D., 2011. Modeling the universal set/reset characteristics of bipolar RRAM by field-and temperature-driven filament growth. *IEEE Transactions on Electron Devices*, *58*(12), pp.4309-4317.

[S33] Ielmini, D, Nardi, F. and Cagli, C., 2011. Physical models of size-dependent nanofilament formation and rupture in NiO resistive switching memories. *Nanotechnology*, *22*(25), p.254022.

[S34] Singh, B. and Surplice, N.A., 1972. The electrical resistivity and resistance-temperature characteristics of thin titanium films. Thin Solid Films, 10(2), pp.243-253.

[S35] F. J. Blatt, Physics of Electron Conduction in Solids. New York: McGraw-Hill, 1968.

[S36] Chen, Z., Huang, W., Zhao, W., Hou, C., Ma, C., Liu, C., Sun, H., Yin, Y. and Li, X., 2019. Ultrafast Multilevel Switching in Au/YIG/n-Si RRAM. Advanced Electronic Materials, 5(2), p.1800418.

[S37] Wang, C., Wu, H., Gao, B., Wu, W., Dai, L., Li, X. and Qian, H., 2017. Ultrafast RESET analysis of HfOx-based RRAM by sub-nanosecond pulses. Advanced Electronic Materials, 3(12), p.1700263.

[S38] Shrestha, P.R., Nminibapiel, D., Kim, J.H., Campbell, J.P., Cheung, K.P., Deora, S., Bersuker, G. and Baumgart, H., 2014, June. Energy control paradigm for compliance-free reliable operation of RRAM. In 2014 IEEE International Reliability Physics Symposium (pp. MY-10). IEEE.

[S39] Choi, B.J., Torrezan, A.C., Strachan, J.P., Kotula, P.G., Lohn, A.J., Marinella, M.J., Li, Z., Williams, R.S. and Yang, J.J., 2016. High-speed and low-energy nitride memristors. Advanced Functional Materials, 26(29), pp.5290-5296.

[S40] Lee, H.Y., Chen, Y.S., Chen, P.S., Gu, P.Y., Hsu, Y.Y., Wang, S.M., Liu, W.H., Tsai, C.H., Sheu, S.S., Chiang, P.C. and Lin, W.P., 2010, December. Evidence and solution of over-RESET problem for HfO x based resistive memory with sub-ns switching speed and high endurance. In 2010 International Electron Devices Meeting (pp. 19-7). IEEE.

[S41] Choi, B.J., Torrezan, A.C., Norris, K.J., Miao, F., Strachan, J.P., Zhang, M.X., Ohlberg, D.A., Kobayashi, N.P., Yang, J.J. and Williams, R.S., 2013. Electrical performance and scalability of Pt dispersed SiO2 nanometallic resistance switch. Nano letters, 13(7), pp.3213-3217.

[S42] Torrezan, A.C., Strachan, J.P., Medeiros-Ribeiro, G. and Williams, R.S., 2011. Sub-nanosecond switching of a tantalum oxide memristor. Nanotechnology, 22(48), p.485203.

[S43] Böttger, U., von Witzleben, M., Havel, V., Fleck, K., Rana, V., Waser, R. and Menzel, S., 2020. Picosecond multilevel resistive switching in tantalum oxide thin films. Scientific reports, 10(1), pp.1-9.

[S44] Zhang, F., Zhang, H., Shrestha, P.R., Zhu, Y., Maize, K., Krylyuk, S., Shakouri, A., Campbell, J.P., Cheung, K.P., Bendersky, L.A. and Davydov, A.V., 2018, December. An ultra-fast multi-level MoTe 2-based RRAM. In 2018 IEEE International Electron Devices Meeting (IEDM) (pp. 22-7). IEEE.

[S45] Ge, R., Wu, X., Kim, M., Chen, P.A., Shi, J., Choi, J., Li, X., Zhang, Y., Chiang, M.H., Lee, J.C. and Akinwande, D., 2018, December. Atomristors: Memory effect in atomically-thin sheets and record RF switches. In 2018 IEEE International Electron Devices Meeting (IEDM) (pp. 22-6). IEEE.

[S46] Kim, M., Ducournau, G., Skrzypczak, S., Yang, S.J., Szriftgiser, P., Wainstein, N., Stern, K., Happy, H., Yalon, E., Pallecchi, E. and Akinwande, D., 2022. Monolayer molybdenum disulfide switches for 6G communication systems. Nature Electronics, pp.1-7.

[S47] Wu, X., Ge, R., Kim, M., Akinwande, D. and Lee, J.C., 2020, February. Atomristors: Non-volatile resistance switching in 2D monolayers. In 2020 Pan Pacific Microelectronics Symposium (Pan Pacific) (pp. 1-6). IEEE.

[S48] Zhang, F., Zhang, H., Krylyuk, S., Milligan, C.A., Zhu, Y., Zemlyanov, D.Y., Bendersky, L.A., Burton, B.P., Davydov, A.V. and Appenzeller, J., 2019. Electric-field induced structural transition in vertical MoTe2-and Mo1–xWxTe2-based resistive memories. Nature materials, 18(1), pp.55-61.

[S49] Veksler, D., Bersuker, G., Bushmaker, A.W., Shrestha, P.R., Cheung, K.P. and Campbell, J.P., 2019, March. Switching variability factors in compliance-free metal oxide RRAM. In 2019 IEEE International Reliability Physics Symposium (IRPS) (pp. 1-5). IEEE.

[S50] Nminibapiel, D.M., Veksler, D., Shrestha, P.R., Campbell, J.P., Ryan, J.T., Baumgart, H. and Cheung, K.P., 2017. Impact of RRAM read fluctuations on the program-verify approach. IEEE Electron Device Letters, 38(6), pp.736-739.

[S51] Wong, H.S.P., Lee, H.Y., Yu, S., Chen, Y.S., Wu, Y., Chen, P.S., Lee, B., Chen, F.T. and Tsai, M.J., 2012. Metal–oxide RRAM. *Proceedings of the IEEE*, *100*(6), pp.1951-1970.

[S52] Ye, C., Wu, J., He, G., Zhang, J., Deng, T., He, P. and Wang, H., 2016. Physical mechanism and performance factors of metal oxide based resistive switching memory: a review. *Journal of Materials Science & Technology*, *32*(1), pp.1-11.

[S53] Kumar, D., Aluguri, R., Chand, U. and Tseng, T.Y., 2017. Metal oxide resistive switching memory: materials, properties and switching mechanisms. *Ceramics International*, *43*, pp.S547-S556.

[S54] Wu, X., Ge, R., Chen, P.A., Chou, H., Zhang, Z., Zhang, Y., Banerjee, S., Chiang, M.H., Lee, J.C. and Akinwande, D., 2019. Thinnest nonvolatile memory based on monolayer h-BN. *Advanced Materials*, *31*(15), p.1806790.

[S55] Kim, M., Ge, R., Wu, X., Lan, X., Tice, J., Lee, J.C. and Akinwande, D., 2018. Zero-static power radio-frequency switches based on MoS2 atomristors. *Nature communications*, *9*(1), p.2524.

[S56] Ge, R., Wu, X., Kim, M., Shi, J., Sonde, S., Tao, L., Zhang, Y., Lee, J.C. and Akinwande, D., 2018. Atomistor: nonvolatile resistance switching in atomic sheets of transition metal dichalcogenides. *Nano letters*, *18*(1), pp.434-441.

[S57] Zhao, H., Dong, Z., Tian, H., DiMarzi, D., Han, M.G., Zhang, L., Yan, X., Liu, F., Shen, L., Han, S.J. and Cronin, S., 2017. Atomically thin femtojoule memristive device. *Advanced Materials*, *29*(47), p.1703232.

[S58] Huang, Y., Gu, Y., Wu, X., Ge, R., Chang, Y.F., Wang, X., Zhang, J., Akinwande, D. and Lee, J.C., 2021. ReSe2-Based RRAM and Circuit-Level Model for Neuromorphic Computing. *Frontiers in Nanotechnology*, *3*, p.782836.

[S59] Xu, R., Jang, H., Lee, M.H., Amanov, D., Cho, Y., Kim, H., Park, S., Shin, H.J. and Ham, D., 2019. Vertical MoS2 double-layer memristor with electrochemical metallization as an atomic-scale synapse with switching thresholds approaching 100 mV. *Nano letters*, *19*(4), pp.2411-2417

[S60] Shi, Y., Pan, C., Chen, V., Raghavan, N., Pey, K.L., Puglisi, F.M., Pop, E., Wong, H.S. and Lanza, M., 2017, December. Coexistence of volatile and non-volatile resistive switching in 2D h-BN based electronic synapses. In *2017 IEEE International Electron Devices Meeting (IEDM)* (pp. 5-4). IEEE.

[S61] Shi, Y., Liang, X., Yuan, B., Chen, V., Li, H., Hui, F., Yu, Z., Yuan, F., Pop, E., Wong, H.S.P. and Lanza, M., 2018. Electronic synapses made of layered two-dimensional materials. *Nature Electronics*, *1*(8), pp.458-465.

[S62] Jing, X., Puglisi, F., Akinwande, D. and Lanza, M., 2019. Chemical vapor deposition of hexagonal boron nitride on metal-coated wafers and transfer-free fabrication of resistive switching devices. *2D Materials*, *6*(3), p.035021.

[S63] Wang, C.H., McClellan, C., Shi, Y., Zheng, X., Chen, V., Lanza, M., Pop, E. and Wong, H.S.P., 2018, December. 3D monolithic stacked 1T1R cells using monolayer MoS 2 FET and hBN RRAM fabricated at low (150° C) temperature. In *2018 IEEE International Electron Devices Meeting (IEDM)* (pp. 22-5). IEEE.

[S64] Yuan, B., Liang, X., Zhong, L., Shi, Y., Palumbo, F., Chen, S., Hui, F., Jing, X., Villena, M.A., Jiang, L. and Lanza, M., 2020. 150 nm× 200 nm cross-point hexagonal boron nitride-based memristors. *Advanced Electronic Materials*, *6*(12), p.1900115.

[S65] Yeh, C.H., Zhang, D., Cao, W. and Banerjee, K., 2020, December. 0.5 T0. 5R-Introducing an Ultra-Compact Memory Cell Enabled by Shared Graphene Edge-Contact and h-BN Insulator. In *2020 IEEE International Electron Devices Meeting (IEDM)* (pp. 12-3). IEEE.

[S66] Gu, Y., Serna, M.I., Mohan, S., Londoño-Calderon, A., Ahmed, T., Huang, Y., Lee, J., Walia, S., Pettes, M.T., Liechti, K.M. and Akinwande, D., 2022. Sulfurization Engineering of One-Step Low-Temperature MoS2 and WS2 Thin Films for Memristor Device Applications. *Advanced Electronic Materials*, *8*(2), p.2100515.

[S67] Shen, Y., Zheng, W., Zhu, K., Xiao, Y., Wen, C., Liu, Y., Jing, X. and Lanza, M., 2021. Variability and Yield in h-BN-Based Memristive Circuits: The Role of Each Type of Defect. *Advanced Materials*, *33*(41), p.2103656.

[S68] Yan, X., Zhao, Q., Chen, A.P., Zhao, J., Zhou, Z., Wang, J., Wang, H., Zhang, L., Li, X., Xiao, Z. and Wang, K., 2019. Vacancy-induced synaptic behavior in 2D WS2 nanosheet–based memristor for low-power neuromorphic computing. *Small*, *15*(24), p.1901423

[S69] Zhuang, P., Lin, W., Ahn, J., Catalano, M., Chou, H., Roy, A., Quevedo-Lopez, M., Colombo, L., Cai, W. and Banerjee, S.K., 2020. Nonpolar resistive switching of multilayer-hBN-based memories. *Advanced Electronic Materials*, *6*(1), p.1900979.

[S70] Tang, B., Veluri, H., Li, Y., Yu, Z.G., Waqar, M., Leong, J.F., Sivan, M., Zamburg, E., Zhang, Y.W., Wang, J. and Thean, A.V., 2022. Wafer-scale solution-processed 2D material analog resistive memory array for memory-based computing. *Nature Communications*, *13*(1), p.3037.

[S71] Wang, M., Cai, S., Pan, C., Wang, C., Lian, X., Zhuo, Y., Xu, K., Cao, T., Pan, X., Wang, B. and Liang, S.J., 2018. Robust memristors based on layered two-dimensional materials. *Nature Electronics*, *1*(2), pp.130-136.

[S72] Braun, D., Lukas, S., Völkel, L., Hartwig, O., Prechtl, M., Belete, M., Kataria, S., Wahlbrink, T., Daus, A., Duesberg, G.S. and Lemme, M.C., 2022, June. Non-Volatile Resistive Switching in PtSe 2-Based Crosspoint Memristors. In *2022 Device Research Conference (DRC)* (pp. 1-2). IEEE.

[S73] Jeong, H.Y., Kim, J.Y., Kim, J.W., Hwang, J.O., Kim, J.E., Lee, J.Y., Yoon, T.H., Cho, B.J., Kim, S.O., Ruoff, R.S. and Choi, S.Y., 2010. Graphene oxide thin films for flexible nonvolatile memory applications. *Nano letters*, *10*(11), pp.4381-4386.

[S74] Wang, Y., Yang, J., Wang, Z., Chen, J., Yang, Q., Lv, Z., Zhou, Y., Zhai, Y., Li, Z. and Han, S.T., 2019. Near-infrared annihilation of conductive filaments in quasiplane MoSe2/Bi2Se3 nanosheets for mimicking heterosynaptic plasticity. *Small*, *15*(7), p.1805431

[S75] Zhu, K., Pazos, S., Aguirre, F., Shen, Y., Yuan, Y., Zheng, W., Alharbi, O., Villena, M.A., Fang, B., Li, X. and Milozzi, A., 2023. Hybrid 2D–CMOS microchips for memristive applications. *Nature*, *618*(7963), pp.57-62.
